# Post-acute and long-term cardiovascular effects following dengue infection: A systematic review protocol

**DOI:** 10.1371/journal.pone.0356838

**Published:** 2026-08-21

**Authors:** Niroshan Chandrajith Lokunarangoda, Akhila Rakshitha Wimalasundera, Manikka H. M. Thisara S. Perera, Damayanthi Idampitiya, H. Naveen Tharaka Perera, K. Gayana Dilki Kothalawala, Arunasalam Pathmeswaran, Namal P. M. Liyanage, Ananda Wijewickrama, U. Arjuna Bandara Medagama

**Affiliations:** 1 Department of Medicine and Mental Health, Faculty of Medicine, University of Moratuwa, Moratuwa, Sri Lanka; 2 Department of Anatomy, Faculty of Medicine, University of Moratuwa, Moratuwa, Sri Lanka; 3 Center for Research and Training, National Institute of Infectious Diseases, Gothatuwa, Sri Lanka; 4 Faculty of Medicine, University of Kelaniya, Kelaniya, Sri Lanka; 5 Department of Microbial Infection and Immunity, College of Medicine, The Ohio State University, Columbus, Ohio, United States of America; 6 Department of Medicine, Faculty of Medicine, University of Peradeniya, Peradeniya, Sri Lanka; Children's National Hospital, George Washington University, UNITED STATES OF AMERICA

## Abstract

**Background and Aim:**

Dengue is a major arboviral disease in tropical and subtropical regions, traditionally regarded as an acute, self-limiting illness. However, emerging evidence suggests that its consequences may extend beyond the acute phase, especially involving the cardiovascular system. Reported manifestations include major adverse cardiovascular events, arrhythmias, thromboembolic complications, subclinical myocardial dysfunction, endothelial dysfunction, and vascular changes. Evidence remains fragmented due to variation in study designs, population characteristics, outcome definitions, and follow-up durations. This systematic review aims to appraise and synthesise evidence on post-acute and long-term cardiovascular outcomes following dengue infection, including their spectrum, comparative risk, and determinants.

**Methods and analysis:**

This protocol outlines a systematic review of cardiovascular outcomes in the post-acute and long-term phases of dengue infection. Human studies of any age group will be included if they report cardiovascular outcomes at least 4 weeks after acute dengue illness. Laboratory-confirmed dengue will be prioritised for primary comparative synthesis; clinically diagnosed dengue will contribute to descriptive synthesis and, where appropriate, subgroup or sensitivity analyses. Studies using cohort, case-control, cross-sectional, or case-series designs will be eligible. Comparative studies with dengue-negative or clearly defined reference controls will estimate effects where available, while non-comparative follow-up studies will contribute to descriptive synthesis. Outcomes will include major clinical cardiovascular events, including stroke or transient ischaemic attacks, subclinical cardiac or vascular abnormalities, and cardiovascular-related biomarkers. Searches will be conducted in PubMed/MEDLINE, Scopus, Cochrane Library, CINAHL, ScienceDirect, ProQuest, and Google Scholar. Two reviewers will independently perform screening, data extraction, and risk-of-bias and critical appraisal assessments. Evidence will be synthesized narratively; where comparable, random-effects meta-analysis and Grading of Recommendations Assessment, Development and Evaluation (GRADE) assessment will be conducted. PROSPERO registration: CRD420261354181.

**Discussion:**

This review will summarize post-dengue cardiovascular sequelae and identify evidence gaps. Findings may clarify post-dengue cardiovascular risk and inform future research, clinical follow-up, and public health planning in dengue-endemic settings.

## Introduction

Dengue is an arboviral disease caused by four dengue virus serotypes (DENV-1 to DENV-4) and transmitted primarily by *Aedes* mosquitoes. It is endemic in more than 100 countries and represents a major public health threat in tropical and subtropical regions, particularly in South and Southeast Asia, Africa, and Latin America [1,2]. Approximately half of the world’s population is now at risk of dengue, with an estimated 100–400 million infections occurring each year [2]. The reported dengue burden has increased substantially over recent decades, with reported cases rising from 505,430 in 2000 to 14.6 million in 2024 [2]. In 2024, the World Health Organization (WHO) received reports of 14,434,584 dengue cases, including 52,738 severe cases and 11,201 deaths across all six WHO regions, marking the highest global reported dengue burden to date [3].

Traditionally, dengue has been considered an acute illness with minimal long-term sequelae. However, emerging epidemiological evidence suggests that dengue infection may be associated with post-acute and longer-term complications affecting multiple organ systems, particularly the cardiovascular system. A population-based cohort study in Taiwan reported a significantly increased risk of major adverse cardiovascular events, including myocardial infarction, stroke, and heart failure, beyond the early period following dengue infection [4]. Another large retrospective cohort study from Singapore reported an increased risk of new-onset cardiovascular disorders within 31–300 days after dengue infection, including dysrhythmias, ischaemic heart disease, and thrombotic events [5]. In addition, observational studies have identified evidence of residual subclinical left ventricular impairment among individuals with previous dengue infection [6].

Although recent observations suggest a potential association, the long-term cardiovascular consequences of dengue infection have not been systematically synthesized. Existing studies vary considerably in terms of design, population characteristics, dengue diagnostic criteria, comparator definitions, outcome definitions, and follow-up duration, resulting in fragmented and inconsistent evidence [1,4–6]. Given the considerable global burden of dengue, a systematic review is necessary to delineate the magnitude and spectrum of post-acute and chronic cardiovascular complications among dengue-exposed populations. Such a review may clarify the diversity of reported cardiovascular outcomes, identify higher-risk subgroups, and guide future epidemiological research and long-term management strategies in dengue-endemic settings.

### Objectives

#### Primary objective.

To systematically review and synthesize available evidence on post-acute and long-term cardiovascular consequences of dengue virus infection, including clinical cardiovascular and cerebrovascular events, subclinical cardiac or vascular changes, and related biomarkers, and to estimate comparative cardiovascular risk where suitable data are available.

#### Specific objectives.

1. To describe the range of post-acute and long-term cardiovascular outcomes reported among individuals with prior dengue infection, including major clinical cardiovascular and cerebrovascular events and subclinical cardiac or vascular abnormalities.
2. To estimate post-acute and long-term cardiovascular risk in dengue-exposed populations compared with non-dengue or general population comparison groups, where comparative data are available.
3. To summarise the evidence on cardiovascular-related biomarkers, including inflammatory, endothelial, and other relevant biomarkers associated with post-acute and long-term cardiovascular risk after dengue infection.
4. To identify reported factors associated with increased post-acute and long-term cardiovascular risk following dengue infection, including patient characteristics, dengue severity, and other reported determinants.

Methodological quality, risk of bias, and heterogeneity across included studies will be assessed and explicitly considered when interpreting findings for all objectives.

#### PROSPERO registration and reporting information.

This protocol is prospectively registered in the International Prospective Register of Systematic Reviews (PROSPERO), registration number CRD420261354181. This protocol was prepared and is reported in accordance with the Preferred Reporting Items for Systematic Review and Meta-Analysis Protocols (PRISMA-P) statement [7,8], and the PRISMA-P checklist is provided in **S1 Checklist**.

#### Protocol amendments.

If important protocol amendments are made after registration, the date, description, and rationale for each amendment will be documented in an amendment log, updated in the PROSPERO record where applicable, and reported in the final systematic review manuscript.

### Planned timeline

The review is planned across four phases. Literature searching and title/abstract screening are planned for June 2026. Full-text review, study selection, data extraction, and risk-of-bias/critical appraisal assessment will take place starting in July 2026. Data synthesis, including narrative synthesis and any meta-analyses, subgroup analyses, or sensitivity analyses, will be conducted from August 2026. Manuscript drafting and submission are planned for October -November 2026. The timeline may be adjusted depending on article retrieval, study volume, and reviewer consensus processes, with regular team meetings used to monitor progress.

### Review question and scope

This review will address two linked questions:

#### Primary comparative question.

Among human participants of any age, is documented dengue infection associated with post-acute and long-term cardiovascular outcomes, compared with individuals without documented recent or index dengue infection or with general-population comparison groups?

#### Secondary descriptive question.

What post-acute and long-term cardiovascular manifestations, including clinical cardiovascular events, subclinical cardiac or vascular abnormalities, and cardiovascular-related biomarkers, have been reported among individuals with prior dengue infection?

For this review, eligible outcomes will be those assessed at least 4 weeks after acute dengue illness or recovery. For synthesis, outcomes will be grouped by timing, where data permit: post-acute outcomes will be considered those assessed from 4 weeks to less than 3 months, and long-term outcomes those assessed at 3 months or later. The 4-week threshold was selected pragmatically to separate post-acute outcomes from acute-phase dengue manifestations and early convalescence. The 3-month threshold was selected to distinguish longer-term follow-up from early post-acute findings. These thresholds are not intended to represent strict biological boundaries. Where studies use different timing definitions or broad follow-up windows, the original definitions will be retained, and timing heterogeneity will be considered in synthesis and interpretation. The review will focus on cardiovascular sequelae after dengue, including clinical events, subclinical abnormalities, and relevant biomarkers. Stroke and transient ischaemic attack (TIA) will be considered within the broader cardiovascular/cerebrovascular outcome domain. Both comparative studies with a non-dengue control or reference group and non-comparative descriptive studies will be included to capture the full range of available evidence.

### Eligibility criteria

Eligibility criteria were defined using a PECOS-informed framework (Population, Exposure, Comparator, Outcomes, and Study design), adapted from the PECO approach recommended for systematic reviews of exposure–health outcome associations, while also allowing the inclusion of non-comparative descriptive studies where relevant [9,10].

#### Population (P).

Human participants of any age, including children, adolescents, adults, and mixed-age populations. Studies restricted to specific age groups will be eligible.

#### Exposure (E).

Documented dengue infection preceding cardiovascular outcome assessment will be eligible. For primary comparative synthesis, laboratory-confirmed dengue will be prioritised, including confirmation by RT-PCR, NS1 antigen testing, viral isolation, seroconversion, or dengue-specific serology, as defined by the original study. Studies based only on clearly stated clinical diagnostic criteria will be included for descriptive synthesis and, where appropriate, subgroup or sensitivity analyses, but will not be pooled with laboratory-confirmed studies unless diagnostic comparability is judged adequate. Primary or secondary dengue infection and any level of acute disease severity will be eligible.

Coinfections and concurrent viral illnesses: Studies reporting dengue with coinfection or concurrent viral illness, including chikungunya, Zika, influenza, SARS-CoV-2, or other arboviruses, will be eligible only when dengue-specific data are reported separately or when coinfection status can be extracted. Where dengue-specific cardiovascular outcome data cannot be separated from mixed-infection cohorts, such studies will not contribute to primary comparative effect estimation and will be summarised narratively only if relevant.

#### Comparator (C).

Eligible comparator groups will include individuals without documented recent or index dengue infection, as defined by the original study. These may include laboratory-confirmed dengue-negative controls, non-dengue febrile illness controls, matched hospital or community controls, population controls, or other clearly defined comparison groups.

For each included comparative study, we will extract how comparator dengue status was established, including molecular, antigen, serological, clinical, diagnostic-code-based, or record-based criteria, as well as absence of recorded or reported dengue history. In dengue-endemic settings, prior asymptomatic dengue infection among comparator participants may not be fully excluded. Therefore, the comparator definition, dengue testing methods, and potential exposure misclassification will be considered during risk-of-bias assessment and interpretation.

Studies without a comparator group will also be eligible if they report relevant post-acute or long-term cardiovascular outcomes among dengue-exposed populations; however, such studies will be used for descriptive synthesis rather than comparative effect estimation.

#### Outcomes (O).

Any post-acute or long-term cardiovascular outcome assessed at least 4 weeks after acute dengue illness or recovery, consistent with the timing framework described above.

The primary outcomes of this review will be post-acute and long-term major clinical cardiovascular events, including cerebrovascular events such as stroke or transient ischaemic attack, particularly those reported in comparative studies with non-dengue or general-population control groups. These will include myocardial infarction or acute coronary syndrome, stroke or transient ischaemic attack, heart failure, clinically documented arrhythmia, myocarditis, cardiomyopathy, thrombotic cardiovascular events, and cardiovascular-related mortality, using definitions reported by the original studies.

Secondary outcomes will include subclinical cardiac or vascular abnormalities, such as electrocardiographic abnormalities, echocardiographic dysfunction, including global longitudinal strain abnormalities, imaging evidence of myocardial or vascular involvement, carotid intima–media thickness, arterial stiffness, endothelial dysfunction, and other reported vascular changes.

Additional exploratory outcomes will include cardiovascular-related biomarkers, such as cardiac troponin, B-type natriuretic peptide (BNP) or N-terminal pro–B-type natriuretic peptide (NT-proBNP), inflammatory markers, coagulation markers, endothelial activation markers, and other laboratory indicators relevant to cardiovascular risk.

Factors associated with increased post-acute or long-term cardiovascular risk, including age, sex, baseline cardiovascular risk factors, dengue severity, primary or secondary infection, serotype, hospitalisation status, follow-up duration, and geographic setting, will also be summarised where reported. Clinical cardiovascular and cerebrovascular outcomes will be prioritised because they have direct clinical relevance and best address the review's primary comparative objective. Subclinical abnormalities and biomarkers will be treated as secondary or exploratory outcomes because they may provide mechanistic or early evidence of cardiovascular involvement but are more likely to be heterogeneous across studies.

#### Study designs (S).

Studies using cohort, case-control, cross-sectional, and case-series designs will be eligible if they report post-acute or long-term cardiovascular outcomes following dengue infection. Comparative studies will be used for effect estimation. Non-comparative follow-up studies, including case series with post-acute follow-up, may be included for descriptive synthesis of the spectrum of outcomes. Case reports, reviews, editorials, commentaries, animal studies, and studies limited exclusively to acute-phase cardiac findings during active dengue illness will not be eligible.

We will include studies published between January 1985 and March 2026. This temporal criterion was selected to capture evidence from the period of modern dengue epidemiology, diagnostic methods, and cardiovascular outcome reporting, while limiting older reports with limited applicability to current diagnostic and follow-up practice. The end date reflects the planned search period for this protocol. Full-text articles published in English, Spanish, or Portuguese will be eligible for inclusion. Potentially relevant reports in other languages will be documented separately but not fully assessed, and this will be acknowledged as a limitation of the review.

### Search strategy

We will perform a comprehensive literature search to identify all relevant studies on post-acute and long-term cardiovascular effects of dengue. The following databases will be searched:

PubMed/MEDLINE, Scopus, Cochrane Library, Cumulative Index to Nursing and Allied Health Literature (CINAHL), ScienceDirect, and ProQuest Dissertations and Theses. We will also search for clinical trial registries, including ClinicalTrials.gov, for relevant completed but unpublished studies. Google Scholar will be used as a supplementary source for grey literature rather than as a primary bibliographic database. Predefined search phrases based on the main concepts of dengue infection, cardiovascular outcomes, and post-acute or long-term follow-up will be used and reported in the search appendix. For each search phrase, up to the first 200 results sorted by relevance will be screened by title and snippet, unless relevance is exhausted earlier (S1 Appendix). Searches will be performed using a consistent browser/account approach where feasible. The search date, search phrases, number of results screened, reason for stopping if fewer than 200 results are screened, and potentially eligible records identified will be documented in a search log.

The search strategy will be developed around three key concepts: (1) dengue infection, (2) cardiovascular or cerebrovascular outcomes, and (3) post-acute and long-term follow-up. We will use a combination of controlled vocabulary and free-text terms, adapted for each database. The full draft PubMed search strategy provided in S1 Appendix will be adapted for use in the other databases. The strategy will be refined through pilot testing and, where feasible, reviewed in consultation with a medical librarian. Both British and American spellings will be used where relevant. Database limits will be restricted to human studies and the publication period from January 1985 to March 2026. No language restrictions will be applied at the search stage where feasible; however, full-text eligibility assessment will be limited to studies published in English, Spanish, or Portuguese. No study-design restrictions will be applied at the search stage. All searches will be documented by database, date, strategy, and number of records retrieved.

Key search terms will include synonyms and variants such as:

- Dengue terms:: “dengue”, “dengue fever”, “severe dengue”, “dengue haemorrhagic fever” (DHF), “dengue virus”, “dengue virus infection”.
- Cardiovascular outcome terms: “cardiovascular disease”, “cardiac”, “heart”, “heart failure”, “cardiomyopathy”, “myocarditis”, “pericarditis”, “arrhythmia”, “atrial fibrillation”, “bradycardia”, “heart block”, “increased carotid intima-media thickness”, “conduction disorder”, “myocardial infarction”, “acute coronary syndrome”, “stroke”, “transient ischaemic attack (TIA)”, “thrombosis”, “thromboembolism”, “major adverse cardiovascular events” (MACE), “atherosclerosis”, “endothelial dysfunction”, “arterial stiffness”, etc.
- Long-term follow-up terms: “long-term”, “chronic”, “post-acute”, “convalescent”, “survivors”, “follow-up studies”, “longitudinal”, “sequelae”, “History.”

These terms will be combined using **AND** between the major concepts (dengue AND cardiovascular AND long-term) and **OR** for synonyms within each concept. Truncation and wildcard symbols will be used where appropriate (e.g., “cardio*” to capture “cardiovascular”, “cardiology”, etc.), and proximity operators will be applied in databases that support them to capture phrases like “cardiovascular complications” near “dengue”.

### Data management

All references retrieved from the searches will be imported into Rayyan for screening and deduplication [11]. Duplicate citations identified automatically by Rayyan will be verified manually before removal. After de-duplication, the reference library will be exported to Zotero for full-text management, long-term storage, citation management, and team-based organisation of records. We will maintain backup copies of the reference database throughout the review.

### Study selection

Before formal screening, the reviewer team will undertake a calibration exercise using a sample of retrieved records to ensure consistent interpretation of the eligibility criteria. Discrepancies will be discussed, and screening guidance will be refined before proceeding with blinded title/abstract screening and independent full-text assessment. Study selection will be conducted in two stages: (1) title/abstract screening and (2) full-text screening. Two reviewers will independently screen all records retrieved from the searches against the eligibility criteria. Rayyan’s blinding function will be used during the initial screening to minimise selection bias [11]. Records judged potentially eligible or unclear by either reviewer will proceed to full-text review. Full texts will then be assessed independently by the same two reviewers. Disagreements at either stage will be resolved by discussion and consensus, with a third reviewer consulted if necessary. Reasons for exclusion at the full-text stage will be documented, and the study selection process will be presented in a PRISMA flow diagram [12]. Where necessary, study authors will be contacted for clarification.

### Data extraction

A standardised electronic data extraction form will be developed and pilot-tested on a small sample of eligible or included studies to ensure clarity, completeness, and consistency. Before formal extraction, reviewers will complete a calibration exercise using this form, compare extracted data, discuss discrepancies, and refine reviewer guidance where necessary. Data extraction will then be performed independently by two reviewers using the electronic extraction system, with discrepancies resolved through discussion and, if necessary, by consultation with a third reviewer.

The data extraction form will capture the following details for each study:

- Publication details: authors, year, journal, country of study.
- Study design: e.g., cohort, case-control, cross-sectional, sample size, duration of follow-up after dengue.
- Population characteristics: age (range/mean), sex distribution, baseline cardiovascular risk factors or comorbidities of participants, dengue severity (e.g., percentage with dengue haemorrhagic fever or requiring hospitalization), dengue serotype if reported, and context (epidemic or endemic setting).
- Exposure details: dengue diagnostic method, including RT-PCR, NS1 antigen testing, viral isolation, seroconversion, dengue-specific serology, or clearly stated clinical diagnostic criteria; timing of diagnosis where reported; primary or secondary dengue status; acute disease severity; and reported coinfections or concurrent viral illnesses, including how these were diagnosed and whether dengue-specific outcome data were reported separately.
- Comparison group: comparator type, source of controls, and characteristics of the control group, where applicable; how comparator dengue status was established, including molecular, antigen, serological, clinical, diagnostic-code-based, or record-based criteria; and any matching, adjustment, or other methods used to address baseline differences between groups.
- Outcomes measured: list of post-acute or long-term cardiovascular outcomes assessed, including clinical events, subclinical measures, and specific biomarkers; outcome definitions used by the original studies; diagnostic or measurement methods; follow-up window; time point(s) of outcome assessment; and reported effect measures.
- Outcome results: numerical results for each relevant outcome. For dichotomous outcomes, we will record event counts and denominators for dengue-exposed and comparator groups where available. For continuous outcomes or biomarker levels, we will record means and standard deviations, medians and interquartile ranges, or other reported summary measures. Adjusted effect estimates, including adjusted hazard ratios, risk ratios, odds ratios, or mean differences from multivariable models, will be extracted preferentially, together with confidence intervals, covariates included in adjustment, and statistical significance where reported.
- Risk-of-bias assessment: We will record the judgments from the risk-of-bias evaluation where necessary
- Other notes: funding source of the study, any reported conflicts of interest, and any other observations (e.g., whether authors mention potential mechanisms or if follow-up was incomplete).

Where multiple reports arise from the same cohort, registry, database, or overlapping population, they will be linked and treated as a single study for each relevant outcome and follow-up timepoint to avoid double counting. The most comprehensive or methodologically appropriate report will be used for primary extraction, with additional reports used only for supplementary non-overlapping information.

Where key information required for eligibility assessment, risk-of-bias assessment, or synthesis is missing or unclear, we will contact study authors where feasible. Unavailable information will be recorded as not reported or unclear. Missing outcome data will not be imputed. Summary statistics will be converted only when methodologically appropriate; otherwise, findings will be summarised narratively. The potential effect of missing data will be considered during risk-of-bias assessment and interpretation.

### Risk-of-bias assessment and critical appraisal

Risk-of-bias and critical appraisal will be assessed independently by two reviewers using study-design-specific tools. For cohort or follow-up studies estimating the effect of dengue exposure on post-acute or long-term cardiovascular outcomes, the Risk Of Bias In Non-randomised Studies—of Exposures tool (ROBINS-E) will be used [13]. ROBINS-E assessments will be performed at the result/outcome level and will consider bias due to confounding, participant selection, exposure classification, departures from intended exposures, missing data, outcome measurement, and selection of the reported result [13]. Missing, incomplete, or unclear outcome and covariate data will be considered when judging risk of bias, including bias due to missing data, and when interpreting the reliability of study findings. Comparator ascertainment, including possible previously undetected dengue infection among controls, will be considered when judging exposure classification bias and interpreting comparative estimates. Prespecified key confounders will include age, sex, hypertension, diabetes, smoking, and other baseline cardiovascular risk factors, where relevant.

For case-control studies, the Joanna Briggs Institute (JBI) Critical Appraisal Checklist for Case Control Studies will be used [14]. For analytical cross-sectional studies, the revised JBI critical appraisal tool for analytical cross-sectional studies will be used [15]. For descriptive evidence, including case series, the relevant JBI critical appraisal checklists will be applied [16]. Disagreements will be resolved through discussion or consultation with a third reviewer where necessary. Risk-of-bias and critical appraisal findings will be summarised in tables and incorporated into the interpretation of the review findings; studies will not be excluded solely based on appraisal results unless serious methodological concerns render the findings uninterpretable.

## Data synthesis and analysis

Given anticipated heterogeneity across studies, the primary synthesis will be narrative. Included studies will be summarised in evidence tables and grouped by outcome category, such as major cardiovascular/cerebrovascular events, subclinical cardiac or vascular abnormalities, and cardiovascular-related biomarkers. Findings will be synthesised qualitatively with attention to the direction, magnitude, and consistency of reported associations, as well as patterns by age, dengue severity, baseline cardiovascular risk, and duration of follow-up.

Where at least two studies are clinically and methodologically comparable in terms of population, dengue exposure definition, comparator type, outcome definition, measurement method, follow-up window, and effect measure, meta-analysis will be considered; otherwise, findings will be synthesised narratively by outcome category. Adjusted estimates will be preferentially pooled where available; unadjusted estimates will be used only when adjusted estimates are unavailable and will not be combined with adjusted estimates unless methodologically appropriate. Time-to-event estimates, such as hazard ratios, will be pooled separately from risk ratios and odds ratios, and different effect measures will not be combined unless conversion is justified.

For dichotomous outcomes, pooled estimates will be expressed as risk ratios, odds ratios, or hazard ratios, as appropriate. For continuous outcomes, mean differences will be used when the same scale is used, and standardised mean differences when comparable constructs are measured using different scales. Random-effects meta-analysis will be used because between-study heterogeneity is expected. The between-study variance will be estimated using the restricted maximum likelihood (REML) estimator. Meta-analysis will be conducted in R using the meta and metafor packages for effect pooling, forest plot generation, and, where appropriate, meta-regression [17–19].

Statistical heterogeneity will be assessed using Cochran’s Q test and the I^2^ statistic [17]. Outcomes with heterogeneous definitions, measurement methods, follow-up windows, comparator groups, or effect measures will not be pooled; instead, they will be summarised narratively by outcome category. Where sufficient studies are available, potential sources of heterogeneity will be explored through predefined subgroup analyses and, if feasible, meta-regression. If at least 10 studies are available for a given meta-analysis, small-study effects and potential publication bias will be explored using funnel plots and Egger’s test. Where meta-analysis is not feasible, findings will be synthesised narratively. Studies with mixed dengue and non-dengue viral infection cohorts will not be included in primary pooled comparative analyses unless dengue-specific outcome data are available; otherwise, they will be summarised narratively where relevant.

Sensitivity analyses will assess the robustness of findings by excluding studies at high or critical risk of bias, varying outcome definitions, restricting analyses to studies with comparator groups, excluding studies without confirmed dengue-negative comparator status, and, where sufficient studies are available, excluding studies with substantial or poorly characterised coinfection. Where feasible, sensitivity analyses will also explore the influence of studies with substantial missing or unclear outcome data. Any material changes in findings will be reported and discussed.

The certainty of evidence for each main outcome will be assessed using the Grading of Recommendations Assessment, Development and Evaluation (GRADE) approach, where appropriate [20]. Certainty will be classified as high, moderate, low, or very low. For observational evidence, certainty will be assessed considering risk of bias, inconsistency, indirectness, imprecision, and publication bias [20]. Upgrading will be considered only where justified by factors such as a large effect size, dose-response gradient, or residual confounding likely reducing rather than increasing the observed association. Summary-of-findings tables will be prepared for the main clinical outcomes where a coherent body of comparative evidence is available. Descriptive evidence, such as case-series evidence, will generally not be subjected to formal GRADE assessment unless it contributes to a clearly defined body of evidence for a main outcome. Because the expected evidence base is likely to consist mainly of observational studies, causal interpretation will be cautious. Residual confounding, exposure and comparator misclassification, differential healthcare contact, surveillance bias, and heterogeneous outcome definitions may influence observed associations. These issues will be considered during risk-of-bias assessment, synthesis, GRADE certainty assessment, and interpretation.

### Subgroup analysis

Where data permit, predefined subgroup analyses will explore whether post-acute or long-term cardiovascular effects differ according to dengue severity, age group, sex, primary versus secondary dengue infection, duration of follow-up, study design, comparator status, and geographic setting. Comparator-related subgroup analyses will categorise studies according to how comparator dengue status was established: laboratory-confirmed dengue-negative controls; controls with no recorded or reported dengue history but without laboratory confirmation of dengue-negative status; and controls with unspecified or unclear dengue status. Where sufficient clinically comparable studies are available, subgroup analyses will compare findings across these comparator categories.

Subgroup analyses will be performed only where sufficient clinically comparable studies are available and will be interpreted cautiously, particularly when based on small numbers of studies or inconsistent subgroup definitions. If studies spanning multiple age groups are included but are clinically heterogeneous and not suitable for meaningful pooling, findings will be synthesised separately by age category.

### Ethical considerations

Ethical approval was not required because this study is a systematic review protocol based on published, publicly accessible literature and does not involve collecting new data from human participants. All included studies will be appropriately cited, and the review will be conducted in accordance with the principles of transparency and good evidence synthesis practice.

### Dissemination plan

Findings will be submitted to a peer-reviewed journal and presented at relevant scientific meetings. Where appropriate, key messages may also be shared with clinicians, public health authorities, or dengue control programmes.

### Declaration of generative AI and AI-assisted technologies in the writing process

During the preparation of this systematic review protocol, the authors used ChatGPT (GPT-5.5 Thinking, OpenAI) to assist with language editing, wording refinement, manuscript organisation, and improvement of clarity and coherence. The tool was not used to generate the research question, determine eligibility criteria, make final methodological decisions, select references, or draw conclusions. All AI-assisted suggestions were critically reviewed, verified, and edited by the authors, who take full responsibility for the accuracy, integrity, originality, and final content of the manuscript.

## Supporting information

S1 Checklist PRISMA-P (preferred reporting items for systematic review and meta-analysis protocols) 2015 checklist: Recommended items to address in a systematic review protocol. (DOCX)

S1 Appendix Search strategy. (DOC)
