# Supplementary material for: Post-acute and long-term cardiovascular effects following dengue infection: A systematic review protocol
Source: PLoS One. 2026 Aug 21;21(8):e0356838. doi: 10.1371/journal.pone.0356838 (PMC13496405; doi:10.1371/journal.pone.0356838)
Supplement: Supplementary file 1 [file pone.0356838.s003.pdf]

PLOS One

Post-acute and long-term cardiovascular effects following dengue infection: a systematic review protocol

--Manuscript Draft--

**Manuscript Number:**	PONE-D-26-25529
**Article Type:**	Study Protocol
**Full Title:**	Post-acute and long-term cardiovascular effects following dengue infection: a systematic review protocol
**Short Title:**	Post-dengue cardiovascular sequelae: a systematic review protocol
**Corresponding Author:**	Niroshan C Lokunarangoda, MD FRCPE FACC<br>University of Moratuwa<br>Moratuwa, SRI LANKA
**Keywords:**	Dengue; cardiovascular disease; cardiovascular outcomes; post-acute sequelae; long-term outcomes; systematic review protocol; Meta-Analysis; biomarkers; cerebrovascular disorders
**Abstract:**	Background and Aim<br>Dengue is a major arboviral disease in tropical and subtropical regions, traditionally regarded as an acute, self-limiting illness. However, emerging evidence suggests that its consequences may extend beyond the acute phase, especially involving the cardiovascular system. Reported manifestations include major adverse cardiovascular events, arrhythmias, thromboembolic complications, subclinical myocardial dysfunction, endothelial dysfunction, and vascular changes. Evidence remains fragmented due to variation in study designs, population characteristics, outcome definitions, and follow-up durations. This systematic review aims to appraise and synthesize evidence on post-acute and long-term cardiovascular outcomes following dengue infection, including their spectrum, comparative risk, and determinants.<br>Methods and analysis<br>This protocol outlines a systematic review of cardiovascular outcomes in the post-acute and long-term phases of dengue infection. Human studies of any age group will be included if they report cardiovascular outcomes at least 4 weeks after acute dengue illness. Eligible designs include cohort, case-control, cross-sectional, and case-series studies. Comparative studies with dengue-negative or general-population controls will estimate effects where available, while non-comparative longitudinal studies will contribute to descriptive synthesis. Searches will be conducted in PubMed/MEDLINE, Scopus, the Cochrane Library, CINAHL, ScienceDirect, ProQuest, and Google Scholar, supplemented by reference list screening and registry searches. Two reviewers will independently perform screening, data extraction, and risk-of-bias or critical-appraisal assessment. The Risk Of Bias In Non-randomized Studies-of Exposures (ROBINS-E) tool will be used for cohort or follow-up studies, and the Joanna Briggs Institute (JBI) tools for case-control, analytical cross-sectional, and case-series studies. Evidence will be synthesized narratively; where studies are clinically and methodologically comparable, random-effects meta-analysis will be conducted. Certainty of evidence will be assessed using the Grading of Recommendations Assessment, Development and Evaluation (GRADE) approach. PROSPERO registration: CRD420261354181.<br>Discussion<br>This review will summarize post-dengue cardiovascular sequelae and identify gaps. Findings may clarify post-dengue cardiovascular risk and inform future research, clinical follow-up, and public health planning in dengue-endemic settings.
**Order of Authors:**	Niroshan Chandrajith Lokunarangoda, MD FRCPE FACC
	Akhila Rakshitha Wimalasundera, MBBS MSC
	Manikka HM Thisara S Perera, MD
	Damayanthi Idampitiya, MD
	H Naveen Tharaka Perera, MBBS

Powered by Editorial Manager® and ProduXion Manager® from Aries Systems Corporation

	K. Gayana Dilki Kothalawala, BAMS
	Arunasalam Pathmeswaran, MD
	Namal P M Liyanage, PhD
	Ananda C Wijewickrama, MD, MRCP
	U Arjuna Bandara Medagama, MD, FRCP
**Additional Information:**	
**Question**	**Response**
**Financial Disclosure**<br><br>Enter a financial disclosure statement that describes the sources of funding for the work included in this submission. Review the submission guidelines for detailed requirements. View published research articles from *PLOS ONE* for specific examples.<br><br>This statement is required for submission and **will appear in the published article** if the submission is accepted. Please make sure it is accurate.<br><br>**Funded studies**<br>Enter a statement with the following details:<br>• Initials of the authors who received each award<br>• Grant numbers awarded to each author<br>• The full name of each funder<br>• URL of each funder website<br>• Did the sponsors or funders play any role in the study design, data collection and analysis, decision to publish, or preparation of the manuscript?<br><br>Did you receive funding for this work?	The author(s) received no specific funding for this work.
**Competing Interests**<br><br>Use the instructions below to enter a competing interest statement for this submission. On behalf of all authors, disclose any competing interests that could be perceived to bias this work—acknowledging all financial support and any other relevant financial or non-financial competing interests.	The authors have declared that no competing interests exist.

Powered by Editorial Manager® and ProduXion Manager® from Aries Systems Corporation

This statement is required for submission and **will appear in the published article** if the submission is accepted. Please make sure it is accurate and that any funding sources listed in your Funding Information later in the submission form are also declared in your Financial Disclosure statement.<br><br>View published research articles from *PLOS ONE* for specific examples.<br><br>**NO authors have competing interests**<br><br>Enter: *The authors have declared that no competing interests exist.*<br><br>**Authors with competing interests**<br><br>Enter competing interest details beginning with this statement:<br><br>*I have read the journal's policy and the authors of this manuscript have the following competing interests: [insert competing interests here]*<br><br>* typeset	
**Ethics Statement**<br><br>Enter an ethics statement for this submission. This statement is required if the study involved:<br><br>• Human participants<br>• Human specimens or tissue<br>• Vertebrate animals or cephalopods<br>• Vertebrate embryos or tissues<br>• Field research<br><br>Write "N/A" if the submission does not require an ethics statement.<br><br>General guidance is provided below. Consult the submission guidelines for detailed instructions. **Make sure that all information entered here is included in the**	N/A

**Methods section of the manuscript.**<br><br>**Format for specific study types**<br><br>**Human Subject Research (involving human participants and/or tissue)**<br>• Give the name of the institutional review board or ethics committee that approved the study<br>• Include the approval number and/or a statement indicating approval of this research<br>• Indicate the form of consent obtained (written/oral) or the reason that consent was not obtained (e.g. the data were analyzed anonymously)<br><br>**Animal Research (involving vertebrate animals, embryos or tissues)**<br>• Provide the name of the Institutional Animal Care and Use Committee (IACUC) or other relevant ethics board that reviewed the study protocol, and indicate whether they approved this research or granted a formal waiver of ethical approval<br>• Include an approval number if one was obtained<br>• If the study involved *non-human primates*, add *additional details* about animal welfare and steps taken to ameliorate suffering<br>• If anesthesia, euthanasia, or any kind of animal sacrifice is part of the study, include briefly which substances and/or methods were applied<br><br>**Field Research**<br><br>Include the following details if this study involves the collection of plant, animal, or other materials from a natural setting:<br>• Field permit number<br>• Name of the institution or relevant body that granted permission	
Data Availability<br><br>Before publication, Authors are required to make fully available and without restriction all data underlying their findings. Sharing the data underlying your findings increases the visibility, credibility, and impact of your work, while supporting transparency and future scientific discovery. Please see our PLOS	No datasets were generated or analysed for this protocol; data extracted during the completed review will be reported in the final article and/or made available as supporting information or from the corresponding author upon reasonable request.

Data Policy page for detailed information on this policy.</p><br><br>A Data Availability Statement, detailing where the data can be accessed, is required at first submission. Insert your Data Availability Statement in the box below.</p><br><br>Please see the data reporting of our submission guidelines for instructions on what you need to include in your Data Availability Statement. Examples of acceptable Data Availability Statements are included here for reference:</p><br> <br> - The minimal data set [and accompanying code, where generated] is available at [repository name] via [DOI hyperlink] <br> - No data was generated by this study. The following existing data sources were used: [dataset name] from [dataset location] available via [web address or DOI hyperlink]. <br> - For Study Protocols: No datasets were generated or analysed during the current study. All relevant data from this study will be made available upon study completion. <br> <br><br><i>PLOS allows rare exemptions to address legal and ethical concerns. If you have legal or ethical restrictions, please use the box below to detail these in full sentences for the Journal team to consider.</i></p><br><br>This statement will be typeset if the manuscript is accepted for publication.</p>	

Powered by Editorial Manager® and ProduXion Manager® from Aries Systems Corporation

Manuscript Click here to access/download;Manuscript;1. Protocol Dengue SysR 28 May 2026.docx

Post-acute and long-term cardiovascular effects following dengue infection: a systematic review protocol

Niroshan Chandrajith Lokunarangoda[1*], Akhila Rakshitha Wimalasundera[2], Manikka H M Thisara S Perera[3], Damayanthi Idampitiya[3], H. Naveen Tharaka Perera[3], K. Gayana Dilki Kothalawala[3], A Pathmeswaran[4], Namal PM Liyanage[5], Ananda Wijewickrama[1], U. Arjuna Bandara Medagama[6]

[1] Department of Medicine and Mental Health, Faculty of Medicine, University of Moratuwa, Sri Lanka

[2] Department of Anatomy, Faculty of Medicine, University of Moratuwa, Sri Lanka

[3] Center for Research and Training, National Institute of Infectious Diseases, Gothatuwa, Sri Lanka

[4] Faculty of Medicine, University of Kelaniya, Sri Lanka

[5] Department of Microbial Infection and Immunity, College of Medicine, The Ohio State University, Ohio, United States

[6] Department of Medicine, Faculty of Medicine, University of Peradeniya, Sri Lanka

*** Corresponding author/guarantor**

Niroshan Chandrajith Lokunarangoda (NCL) E-mail: niroshanl@uom.lk (NCL)

ORCID ID: 0000-0001-8216-8381

Abstract

Background and Aim

Dengue is a major arboviral disease in tropical and subtropical regions, traditionally regarded as an acute, self-limiting illness. However, emerging evidence suggests that its consequences may extend beyond the acute phase, especially involving the cardiovascular system. Reported manifestations include major adverse cardiovascular events, arrhythmias, thromboembolic complications, subclinical myocardial dysfunction, endothelial dysfunction, and vascular changes. Evidence remains fragmented due to variation in study designs, population characteristics, outcome definitions, and follow-up durations. This systematic review aims to appraise and synthesize evidence on post-acute and long-term cardiovascular outcomes following dengue infection, including their spectrum, comparative risk, and determinants.

Methods and analysis

This protocol outlines a systematic review of cardiovascular outcomes in the post-acute and long-term phases of dengue infection. Human studies of any age group will be included if they report cardiovascular outcomes at least 4 weeks after acute dengue illness. Eligible designs include cohort, case-control, cross-sectional, and case-series studies. Comparative studies with dengue-negative or general-population controls will estimate effects where available, while non-comparative longitudinal studies will contribute to descriptive synthesis. Searches will be conducted in PubMed/MEDLINE, Scopus, the Cochrane Library, CINAHL, ScienceDirect, ProQuest, and Google Scholar, supplemented by reference list screening and registry searches. Two reviewers will independently perform screening, data extraction, and

Reviewer: Clarify exposure definition.

risk-of-bias or critical-appraisal assessment. The Risk Of Bias In Non-randomized Studies-of Exposures (ROBINS-E) tool will be used for cohort or follow-up studies, and the Joanna Briggs Institute (JBI) tools for case-control, analytical cross-sectional, and case-series studies. Evidence will be synthesized narratively; where studies are clinically and methodologically comparable, random-effects meta-analysis will be conducted. Certainty of evidence will be assessed using the Grading of Recommendations Assessment, Development and Evaluation (GRADE) approach. PROSPERO registration: CRD420261354181.

Discussion

This review will summarize post-dengue cardiovascular sequelae and identify gaps. Findings may clarify post-dengue cardiovascular risk and inform future research, clinical follow-up, and public health planning in dengue-endemic settings.

Introduction

Dengue is an arboviral disease caused by four dengue virus serotypes (DENV-1 to DENV-4) and transmitted primarily by *Aedes* mosquitoes. It is endemic in more than 100 countries and represents a major public health threat in tropical and subtropical regions, particularly in South and Southeast Asia, Africa, and Latin America [1,2]. Approximately half of the world's population is now at risk of dengue, with an estimated 100–400 million infections occurring each year [2]. The reported dengue

Reviewer: Cite fragmentation; improve eligibility wording.

burden has increased substantially over recent decades, with reported cases rising from 505,430 in 2000 to 14.6 million in 2024 [2]. In 2024, the World Health Organization (WHO) received reports of 14,434,584 dengue cases, including 52,738 severe cases and 11,201 deaths across all six WHO regions, marking the highest global reported dengue burden to date [3].

Traditionally, dengue has been considered an acute illness with minimal long-term sequelae. However, emerging epidemiological evidence suggests that dengue infection may be associated with post-acute and longer-term complications affecting multiple organ systems, particularly the cardiovascular system. A population-based cohort study in Taiwan reported a significantly increased risk of major adverse cardiovascular events, including myocardial infarction, stroke, and heart failure, beyond the early period following dengue infection [4]. Another large retrospective cohort study from Singapore reported an increased risk of new-onset cardiovascular disorders within 31–300 days after dengue infection, including dysrhythmias, ischaemic heart disease, and thrombotic events [5]. In addition, observational studies have identified evidence of residual subclinical left ventricular impairment among individuals with previous dengue infection [6].

Although recent observations suggest a potential association, the long-term cardiovascular consequences of dengue infection have not been systematically synthesized. Existing studies vary considerably in terms of design, population characteristics, outcome definitions, and follow-up duration, resulting in fragmented and inconsistent evidence. Given the considerable global burden of dengue, a systematic review is necessary to delineate the magnitude and spectrum of post-acute

and chronic cardiovascular complications among dengue-exposed populations. Such a review may clarify the diversity of reported cardiovascular outcomes, identify higher-risk subgroups, and guide future epidemiological research and long-term management strategies in dengue-endemic settings.

Objectives

Primary Objective: To systematically review and synthesize all available evidence on post-acute and long-term cardiovascular consequences of dengue virus infection, including clinical cardiovascular events, subclinical cardiac or vascular changes, and related biomarkers, to determine the magnitude and determinants of post-dengue cardiovascular risk.

Specific Objectives

1. To describe the range of post-acute and long-term cardiovascular outcomes reported among individuals with prior dengue infection, including major clinical cardiovascular events and subclinical cardiac or vascular abnormalities.
2. To compare post-acute and long-term cardiovascular risk in dengue-exposed populations with that in non-dengue or general population comparison groups, where comparative data are available.
3. To summarise the evidence on cardiovascular-related biomarkers, including inflammatory, endothelial, and other relevant biomarkers associated with post-acute and long-term cardiovascular risk after dengue infection.

Reviewer: Avoid overstatement in objectives.

4. To identify factors associated with increased post-acute and long-term cardiovascular risk following dengue infection, including patient characteristics, dengue severity, and other reported determinants.

Methodological quality, risk of bias, and heterogeneity across included studies will be assessed and explicitly considered when interpreting findings for all objectives.

PROSPERO Registration and reporting information: This protocol is prospectively registered in the International Prospective Register of Systematic Reviews (PROSPERO), registration number CRD420261354181. This protocol was prepared and is reported in accordance with the Preferred Reporting Items for Systematic Review and Meta-Analysis Protocols (PRISMA-P) statement [7,8], and the PRISMA-P checklist is provided in **S1 Checklist.**

Protocol amendments: If important protocol amendments are made after registration, the date, description, and rationale for each amendment will be documented in an amendment log, updated in the PROSPERO record where applicable, and reported in the final systematic review manuscript.

Planned Timeline

The review is planned across four phases. Literature searching and title/abstract screening are planned for June 2026. Full-text review, study selection, data extraction, and risk-of-bias/critical appraisal assessment will take place starting in July 2026. Data synthesis, including narrative synthesis and any meta-analysis, subgroup analyses, or sensitivity analyses, will be conducted from August 2026. Manuscript drafting and submission are planned for October -November 2026. The timeline may

be adjusted depending on article retrieval, study volume, and reviewer consensus processes, with regular team meetings used to monitor progress.

Review Question and Scope

This review will address two linked questions:

Primary comparative question:

Among human participants of any age, is documented dengue infection associated with post-acute and long-term cardiovascular outcomes, compared with individuals without documented recent or index dengue infection or with general-population comparison groups?

Secondary descriptive question:

What post-acute and long-term cardiovascular manifestations, including clinical cardiovascular events, subclinical cardiac or vascular abnormalities, and cardiovascular-related biomarkers, have been reported among individuals with prior dengue infection?

For this review, eligible outcomes will be those assessed at least 4 weeks after acute dengue illness or recovery. For synthesis, outcomes will be grouped by timing, where data permit: post-acute outcomes will be considered those assessed from 4 weeks to less than 3 months, and long-term outcomes those assessed at 3 months or later. Where studies use different cut-offs or report broad follow-up windows, the original study definitions will be retained, and timing differences will be considered when

interpreting findings. The review will focus on cardiovascular sequelae after dengue, including clinical events, subclinical abnormalities, and relevant biomarkers. Stroke and transient ischaemic attack (TIA) will be considered within the broader cardiovascular/cerebrovascular outcome domain. Both comparative studies with a non-dengue control or reference group and non-comparative descriptive studies will be included to capture the full range of available evidence.

Eligibility Criteria

Eligibility criteria were defined using a PECOS-informed framework (Population, Exposure, Comparator, Outcomes, and Study design), adapted from the PECO approach recommended for systematic reviews of exposure–health outcome associations, while also allowing the inclusion of non-comparative descriptive studies where relevant [9,10].

Population (P):

Human participants of any age, including children, adolescents, adults, and mixed-age populations. Studies restricted to specific age groups will be eligible.

Exposure (E):

Documented dengue infection preceding cardiovascular outcome assessment, confirmed by laboratory, serological, molecular, or clearly stated clinical diagnostic criteria, as defined by the original study. Studies involving primary or secondary dengue infection, and any level of acute disease severity, will be eligible.

Comparator (C):

Reviewer: Justify post-acute cutoffs.

Individuals without documented recent or index dengue infection, including dengue-negative controls, non-dengue febrile illness controls, population controls, matched controls, or other clearly defined comparison groups, where available. In dengue-endemic settings, the absence of any previous dengue infection may not be fully ascertainable; therefore, comparator definitions, dengue testing methods, and potential exposure misclassification will be extracted and considered in the risk-of-bias assessment and interpretation. Studies without a comparator group will also be eligible if they report relevant post-acute or long-term cardiovascular outcomes among dengue-exposed populations; however, such studies will be used for descriptive synthesis rather than comparative effect estimation.

Outcomes (O):

Any post-acute or long-term cardiovascular outcome assessed at least 4 weeks after acute dengue illness.

The primary outcomes of this review will be post-acute and long-term clinical cardiovascular and cerebrovascular events following dengue infection, particularly those reported in comparative studies with non-dengue or general-population control groups. These will include myocardial infarction or acute coronary syndrome, stroke or transient ischaemic attack, heart failure, arrhythmia, myocarditis, cardiomyopathy, thrombotic cardiovascular events, and cardiovascular-related mortality.

Secondary outcomes will include subclinical cardiac or vascular abnormalities, such as electrocardiographic abnormalities, echocardiographic dysfunction, global longitudinal strain abnormalities, imaging evidence of myocardial or vascular

Reviewer: Clarify comparator misclassification.

involvement, carotid intima–media thickness, arterial stiffness, endothelial dysfunction, and other reported vascular changes.

Additional exploratory outcomes will include cardiovascular-related biomarkers, such as cardiac troponin, B-type natriuretic peptide (BNP) or N-terminal pro–B-type natriuretic peptide (NT-proBNP), inflammatory markers, coagulation markers, endothelial activation markers, and other laboratory indicators relevant to cardiovascular risk. Factors associated with increased post-acute or long-term cardiovascular risk, including age, sex, baseline cardiovascular risk factors, dengue severity, primary or secondary infection, serotype, hospitalisation status, follow-up duration, and geographic setting, will also be summarised where reported.

Clinical cardiovascular and cerebrovascular outcomes will be prioritised because they have direct clinical relevance and best address the review's primary comparative objective. Subclinical abnormalities and biomarkers will be treated as secondary or exploratory outcomes because they may provide mechanistic or early evidence of cardiovascular involvement but are more likely to be heterogeneous across studies.

Study designs (S):

Eligible studies will include cohort, case-control, cross-sectional, and case-series studies reporting post-acute or long-term cardiovascular outcomes following dengue. Comparative studies will be used for effect estimation. Non-comparative follow-up studies, including case series with post-acute follow-up, may be included for descriptive synthesis of the spectrum of outcomes. Case reports, reviews, editorials,

commentaries, animal studies, and studies limited exclusively to acute-phase cardiac findings during active dengue illness will not be eligible.

We will include studies published between January 1985 and March 2026. Only studies published in English or Spanish will be included. Portuguese-language studies identified during screening will be listed separately as potentially relevant but not fully assessed, and this will be acknowledged as a limitation.

Search Strategy

We will perform a comprehensive literature search to identify all relevant studies on post-acute and long-term cardiovascular effects of dengue. The following databases will be searched:

PubMed/MEDLINE, Scopus, Cochrane Library, Cumulative Index to Nursing and Allied Health Literature (CINAHL), ScienceDirect, and ProQuest Dissertations and Theses. Google Scholar will be searched for additional grey literature, and the reference lists of included studies and relevant reviews will be manually screened to identify additional eligible reports. We will also search for clinical trial registries, including ClinicalTrials.gov, for relevant completed but unpublished studies.

The search strategy will be developed around three key concepts: (1) dengue infection, (2) cardiovascular or cerebrovascular outcomes, and (3) post-acute and long-term follow-up. We will use a combination of controlled vocabulary and free-text terms, adapted for each database. The full draft PubMed search strategy provided in S1 Appendix will be adapted for use in the other databases. The strategy will be refined through pilot testing and, where feasible, reviewed in consultation with a medical librarian. Both British and American spellings will be used where relevant. Database

limits will be restricted to human studies and the publication period January 1985 to March 2026. No study-design restrictions will be applied at the search stage. All searches will be documented by database, date, strategy, and number of records retrieved.

Key search terms will include synonyms and variants such as:

- **Dengue terms:** “dengue”, “dengue fever”, “severe dengue”, “dengue haemorrhagic fever” (DHF), "dengue virus”, “dengue virus infection”.
- **Cardiovascular outcome terms:** “cardiovascular disease”, “cardiac”, “heart”, “heart failure”, “cardiomyopathy”, “myocarditis”, “pericarditis”, “arrhythmia”, “atrial fibrillation”, “bradycardia”, “heart block”, “increased carotid intima-media thickness”, “conduction disorder”, “myocardial infarction”, “acute coronary syndrome”, “stroke”, “transient ischaemic attack (TIA)”, “thrombosis”, “thromboembolism”, “major adverse cardiovascular events” (MACE), “atherosclerosis”, “endothelial dysfunction”, “arterial stiffness”, etc.
- **Long-term follow-up terms:** “long-term”, “chronic”, “post-acute”, “convalescent”, “survivors”, “follow-up studies”, “longitudinal”, “sequelae”, “History.”

These terms will be combined using **AND** between the major concepts (dengue AND cardiovascular AND long-term) and **OR** for synonyms within each concept. Truncation and wildcard symbols will be used where appropriate (e.g., “cardio*” to capture “cardiovascular”, “cardiology”, etc.), and proximity operators will be applied

Reviewer: Justify language restriction.

in databases that support them to capture phrases like "cardiovascular complications" near "dengue".

Data Management

All references retrieved from the searches will be imported into Rayyan for screening and deduplication [11]. Duplicate citations identified automatically by Rayyan will be verified manually before removal. After de-duplication, the reference library will be exported to Zotero for full-text management, long-term storage, citation management, and team-based organisation of records. We will maintain backup copies of the reference database throughout the review.

Study Selection

Study selection will be conducted in two stages: (1) title/abstract screening and (2) full-text screening. Two reviewers will independently screen all records retrieved from the searches against the eligibility criteria. Rayyan's blinding function will be used during the initial screening to minimise selection bias [11]. Records judged potentially eligible or unclear by either reviewer will proceed to full-text review. Full texts will then be assessed independently by the same two reviewers. Disagreements at either stage will be resolved by discussion and consensus, with a third reviewer consulted if necessary. Reasons for exclusion at the full-text stage will be documented, and the study selection process will be presented in a PRISMA flow diagram [12]. Where necessary, study authors will be contacted for clarification.

Reviewer: Define Google Scholar methodology.

Data Extraction

A standardised electronic data extraction form will be developed and pilot-tested on a small sample of included studies to ensure clarity, completeness, and consistency. Data extraction will be performed independently by two reviewers using the electronic extraction system, with discrepancies resolved through discussion and, if necessary, by consultation with a third reviewer.

The data extraction form will capture the following details for each study:

- Publication details: authors, year, journal, country of study.
- Study design: e.g., cohort, case-control, cross-sectional, sample size, duration of follow-up after dengue.
- Population characteristics: age (range/mean), sex distribution, baseline cardiovascular risk factors or comorbidities of participants, dengue severity (e.g., percentage with dengue haemorrhagic fever or requiring hospitalization), dengue serotype if reported, and context (epidemic or endemic setting).
- Exposure details: confirmation method for dengue infection (e.g. serology, polymerase chain reaction-PCR), whether primary or secondary dengue (if reported).
- Comparison group: characteristics of control group (if any) – source of controls, matching or adjustments done, etc.
- Outcomes measured: list of long-term cardiovascular outcomes assessed (clinical events, any subclinical measures, specific biomarkers) and the time point(s) of outcome assessment

- Outcome results: numerical results for each relevant outcome. For dichotomous outcomes (e.g. incidence of myocardial infarction), we will record the number of events and total in dengue survivors and controls (to derive risk ratios or odds ratios); for continuous outcomes or biomarker levels, we will record mean values and standard deviations or other effect measures (e.g. mean difference, or median with interquartile range if only non-parametric data given). If adjusted effect estimates (e.g., adjusted hazard ratio or odds ratio comparing dengue vs no dengue) are reported from multivariable models, those will be extracted preferentially. We will also note any statistical significance and confidence intervals.
- Risk-of-bias assessment: We will record the judgments from the risk-of-bias evaluation where necessary
- Other notes: funding source of the study, any reported conflicts of interest, and any other observations (e.g., whether authors mention potential mechanisms or if follow-up was incomplete).

If multiple publications report results from the same cohort, we will extract data from all publications and treat them as a single study (choosing the most comprehensive or latest dataset as the primary dataset and using the others for supplemental information).

Risk-of-Bias Assessment and Critical Appraisal

The risk-of-bias assessment and critical appraisal will be assessed independently by two reviewers using study-design-specific tools. For cohort or follow-up studies estimating the effect of dengue exposure on post-acute or long-term cardiovascular

Reviewer: Specify effect pooling hierarchy.

outcomes, the Risk Of Bias In Non-randomised Studies—of Exposures tool (ROBINS-E) will be used [13]. ROBINS-E assessments will be performed at the result/outcome level and will consider bias due to confounding, participant selection, exposure classification, departures from intended exposures, missing data, outcome measurement, and selection of the reported result [13]. Prespecified key confounders will include age, sex, hypertension, diabetes, smoking, and other baseline cardiovascular risk factors, where relevant.

For case-control studies, the Joanna Briggs Institute (JBI) Critical Appraisal Checklist for Case Control Studies will be used [14]. For analytical cross-sectional studies, the revised JBI critical appraisal tool for analytical cross-sectional studies will be used [15]. For descriptive evidence, including case series, the relevant JBI critical appraisal checklists will be applied [16]. Disagreements will be resolved through discussion or consultation with a third reviewer where necessary. Risk-of-bias and critical appraisal findings will be summarised in tables and incorporated into the interpretation of the review findings; studies will not be excluded solely based on appraisal results unless serious methodological concerns render the findings uninterpretable.

Data Synthesis and Analysis

Given anticipated heterogeneity across studies, the primary synthesis will be narrative. Included studies will be summarised in evidence tables and grouped by outcome category, such as major cardiovascular/cerebrovascular events, subclinical cardiac or

Reviewer: Add reviewer calibration.

vascular abnormalities, and cardiovascular-related biomarkers. Findings will be synthesised qualitatively with attention to the direction, magnitude, and consistency of reported associations, as well as patterns by age, dengue severity, baseline cardiovascular risk, and duration of follow-up.

Where a sufficient number of studies are clinically and methodologically comparable, a random-effects meta-analysis will be conducted, as between-study heterogeneity is expected. Meta-analysis will be conducted in R using the meta and metafor packages for effect pooling, forest plot generation, and, where appropriate, meta-regression [17–19]. For dichotomous outcomes, pooled effect estimates will be expressed as odds ratios, risk ratios, or hazard ratios, as appropriate; for continuous outcomes, mean differences or standardised mean differences will be used. All pooled estimates will be presented with 95% confidence intervals.

Statistical heterogeneity will be assessed using Cochran's Q test and the I^2 statistic [17]. Where sufficient studies are available, potential sources of heterogeneity will be explored through predefined subgroup analyses and, if feasible, meta-regression. If at least 10 studies are available for a given meta-analysis, small-study effects and potential publication bias will be explored using funnel plots and Egger's test. Where meta-analysis is not feasible, findings will be synthesised narratively.

Sensitivity analyses will assess the robustness of findings by excluding studies at high or critical risk of bias, varying outcome definitions, and, where relevant, restricting

analyses to studies with comparator groups. Any material changes in findings will be reported and discussed.

The certainty of evidence for each main outcome will be assessed using the Grading of Recommendations Assessment, Development and Evaluation approach, where appropriate [20]. Certainty will be classified as high, moderate, low, or very low, based on consideration of risk of bias, inconsistency, indirectness, imprecision, and publication bias [20]. For descriptive evidence, such as case series, findings will be summarised narratively and generally not subjected to formal GRADE assessment unless they contribute to a clearly defined body of evidence for a main outcome.

Subgroup Analysis

Where data permit, predefined subgroup analyses will explore whether post-acute or long-term cardiovascular effects differ according to dengue severity, age group, sex, primary versus secondary dengue infection, duration of follow-up, study design, comparator status, and geographic setting. Subgroup analyses will be performed only where sufficient clinically comparable studies are available and will be interpreted cautiously, particularly when based on small numbers of studies or inconsistent subgroup definitions. If studies spanning multiple age groups are included but are clinically heterogeneous and not suitable for meaningful pooling, findings will be synthesised separately by age category.

Ethical Considerations

Ethical approval was not required because this study is a systematic review protocol based on published, publicly accessible literature and does not involve collecting new data from human participants. All included studies will be appropriately cited, and the

Reviewer: Specify random-effects estimator and GRADE details.

review will be conducted in accordance with the principles of transparency and good evidence synthesis practice.

Dissemination Plan

Findings will be submitted to a peer-reviewed journal and presented at relevant scientific meetings. Where appropriate, key messages may also be shared with clinicians, public health authorities, or dengue control programmes.

Conflicts of Interest

The authors declare no conflicts of interest. No financial, personal, or institutional relationships are considered to have influenced this review.

Funding

The authors received no specific funding for this study. The review will be conducted using the authors' own institutional and personal resources. No funder or sponsor had, or will have, any role in the conception, design, drafting, conduct, data collection, analysis, interpretation, manuscript preparation, or decision to submit the final review for publication.

Data Availability Statement

No datasets were generated or analysed during the current protocol study. All relevant data generated during the completed systematic review will be made available upon study completion, in accordance with PLOS ONE data availability requirements.

Author contributions

Conceptualization: Niroshan Chandrajith Lokunarangoda (NCL).

Methodology: NCL, Akhila Rakshitha Wimalasundera (ARW), Manikka H M Thisara S Perera (MTP), Damayanthi Idampitiya (DID), H. Naveen Tharaka Perera (NTP), K. Gayana Dilki Kothalawala (KGD), A Pathmeswaran (APW), Namal PM Liyanage (NML), Ananda Wijewickrama (ADW), and U. Arjuna Bandara Medagama (ABM).

Project administration: NCL, MTP, and DID.

Writing – original draft: NCL.

Writing – review & editing: ARW, MTP, DID, NTP, KGD, APW, NML, ADW, and ABM.

Supervision: APW, NML, ADW, and ABM.

All authors have read and approved the final manuscript and agree to be accountable for all aspects of the work.

Declaration of generative AI and AI-assisted technologies in the writing process

During the preparation of this systematic review protocol, the authors used ChatGPT (GPT-5.5 Thinking, OpenAI) to assist with language editing, wording refinement, manuscript organisation, and improvement of clarity and coherence. The tool was not used to generate the research question, determine eligibility criteria, make final methodological decisions, select references, or draw conclusions. All AI-assisted suggestions were critically reviewed, verified, and edited by the authors, who take full responsibility for the accuracy, integrity, originality, and final content of the manuscript.

References

1. Rahim A, Hameed A, Ishaq U, Malik J, Zaidi SMJ, Khurshid H, et al. Cardiovascular sequelae of dengue fever: a systematic review. Expert Rev Cardiovasc Ther. 2022;20: 465–479. doi:10.1080/14779072.2022.2082945

2. World Health Organization. Dengue and severe dengue. Geneva: World Health Organization; 2025 Aug 21 [cited 2026 May 19]. Available from: https://www.who.int/news-room/fact-sheets/detail/dengue-and-severe-dengue?utm_source

3. World Health Organization. Dengue: global situation, surveillance and progress – 2024 update. Wkly Epidemiol Rec. 2025;100(52):665–678. Available from: https://www.who.int/publications/i/item/who-wer10052-665-678?utm_source

4. Wei KC, Sy CL, Wang WH, Wu CL, Chang S, Huang YT. Major acute cardiovascular events after dengue infection–A population-based observational study. PLoS Negl Trop Dis. 2022;16. doi:10.1371/journal.pntd.0010134

5. Wee LE, Lim JT, Tan JYJ, Malek MIBA, Chiew C, Ng LC, et al. Dengue versus COVID-19: comparing the incidence of cardiovascular, neuropsychiatric and autoimmune complications. J Travel Med. 2024;31. doi:10.1093/jtm/taae081

6. Kaagaard MD, Wegener A, Gomes LC, Holm AE, Lima KO, Matos LO, et al. Potential role of transthoracic echocardiography for screening LV systolic dysfunction in patients with a history of dengue infection. A cross-sectional and cohort study and review of the literature. PLoS ONE. Public Library of Science; 2022. doi:10.1371/journal.pone.0276725

7. Moher D, Shamseer L, Clarke M, Ghersi D, Liberati A, Petticrew M, et al. Preferred reporting items for systematic review and meta-analysis protocols (PRISMA-P) 2015 statement. Syst Rev. 2015;4(1):1. doi:10.1186/2046-4053-4-1.

8. Shamseer L, Moher D, Clarke M, Ghersi D, Liberati A, Petticrew M, et al. Preferred reporting items for systematic review and meta-analysis protocols (prisma-p) 2015: Elaboration and explanation. BMJ (Online). BMJ Publishing Group; 2015. doi:10.1136/bmj.g7647

9. Pérez Velasco R, Jarosińska D. Update of the WHO global air quality guidelines: Systematic reviews – An introduction. Environ Int. 2022;170. doi:10.1016/j.envint.2022.107556

10. Morgan RL, Whaley P, Thayer KA, Schünemann HJ. Identifying the PECO: A framework for formulating good questions to explore the association of environmental and other exposures with health outcomes. Environment International. Elsevier Ltd; 2018. pp. 1027–1031. doi:10.1016/j.envint.2018.07.015

11. Ouzzani M, Hammady H, Fedorowicz Z, Elmagarmid A. Rayyan—a web and mobile app for systematic reviews. Syst Rev. 2016;5: 210. doi:10.1186/s13643-016-0384-4

12. Page MJ, McKenzie JE, Bossuyt PM, Boutron I, Hoffmann TC, Mulrow CD, et al. The PRISMA 2020 statement: an updated guideline for reporting systematic reviews. BMJ. 2021; n71. doi:10.1136/bmj.n71

13. Higgins JPT, Morgan RL, Rooney AA, Taylor KW, Thayer KA, Silva RA, et al. A tool to assess risk of bias in non-randomized follow-up studies of exposure effects (ROBINS-E). Environ Int. 2024;186. doi:10.1016/j.envint.2024.108602

14. Joanna Briggs Institute. JBI critical appraisal checklist for case control studies. Adelaide: Joanna Briggs Institute; 2020. Available from: https://jbi.global/sites/default/files/2020-08/Checklist_for_Case_Control_Studies.pdf?utm_source

15. Barker TH, Hasanoff S, Aromataris E, Stone JC, Leonardi-Bee J, Sears K, et al. The revised JBI critical appraisal tool for the assessment of risk of bias for analytical cross-sectional studies. JBI Evid Synth. 2026;24(3):401–408. doi: 10.11124/JBIES-24-00523.

16. Munn Z; Barker TH; Moola S; Tufanaru C; Stern C; McArthur A; Stephenson M; Aromataris E. Methodological quality of case series studies: an introduction to the JBI critical appraisal tool. JBI Evid Synth. 2020;18: 2127–2133.

17. Duan Y, Mathew T, Alemayehu D, Cheng G. Novel approaches for random-effects meta-analysis of a small number of studies under normality. Res Synth Methods. 2025. doi:10.1017/rsm.2025.10022

18. Balduzzi S, Rücker G, Schwarzer G. How to perform a meta-analysis with R: a practical tutorial. Evid Based Ment Health. 2019;22:153–160. doi:10.1136/ebmental-2019-300117.

19. Viechtbauer W. Conducting meta-analyses in R with the metafor package. J Stat Softw. 2010;36:1–48. doi:10.18637/jss.v036.i03.

20. Guyatt G, Oxman AD, Akl EA, Kunz R, Vist G, Brozek J, et al. GRADE guidelines: 1. Introduction - GRADE evidence profiles and summary of findings tables. J Clin Epidemiol. 2011;64: 383–394. doi:10.1016/j.jclinepi.2010.04.026

…………………………………………………………………………………….

Supporting information

S1 Checklist. PRISMA-P (Preferred Reporting Items for Systematic Review and Meta-Analysis Protocols) 2015 checklist: recommended items to address in a systematic review protocol.

(PDF)

S1 Appendix: PubMed database search strategy

(DOCX)

………………………………………………………………………………..

Review Team and Roles

The review will be conducted by a multidisciplinary team with expertise in epidemiology, cardiology, infectious diseases, and evidence synthesis. **NCL** is the guarantor, conceived the review, led protocol development, and will oversee all stages. **KGD, ARW, NTP, APW, MTP, DID, and ABM** will undertake literature searching, screening, data extraction, risk-of-bias assessment, and statistical analysis, including meta-analysis where feasible. **NML, ADW, APW, MTP, DID, and ABM** will contribute to data synthesis, subgroup and sensitivity analyses, interpretation, and manuscript drafting. All authors will contribute to writing, critically revise the manuscript, approve the final version, and share responsibility for the integrity of the review.

Author Details

1) NCL – Prof. Niroshan Chandrajith Lokunarangoda – niroshanl@uom.lk

(Department of Medicine and Mental Health, Faculty of Medicine, University of Moratuwa, Sri Lanka)

ORCID ID: 0000-0001-8216-8381

2) ARW – Dr Akhila Rakshitha Wimalasundera – akhilaw@uom.lk

(Department of Anatomy, Faculty of Medicine, University of Moratuwa, Sri Lanka) ORCID ID: 0000-0002-4209-1057

3) MTP- Dr Manikka H M Thisara S Perera - thisaraperera7@gmail.com

(Center for Research and Training (CeRT), National Institute of Infectious Diseases (NIID), Gothatuwa, Sri Lanka)

ORCID ID: 0009-0007-2291-8356

4) DID- Dr Damayanthi Idampitiya - damayanthi_idampitiya@yahoo.com

(Center for Research and Training (CeRT), National Institute of Infectious Diseases (NIID), Gothatuwa, Sri Lanka)

ORCID ID: 0000-0003-2938-5656

5) NTP – Dr H. Naveen Tharaka Perera – naveentharakacr7@gmail.com

(Center for Research and Training (CeRT), National Institute of Infectious Diseases (NIID), Gothatuwa, Sri Lanka)

ORCID ID: 0009-0007-0250-7082

6) KGD – Dr K. Gayana Dilki Kothalawala – gayanadilki123@gmail.com

(Center for Research and Training (CeRT), National Institute of Infectious Diseases (NIID), Gothatuwa, Sri Lanka)
ORCID ID 0009-0001-4993-9129

7) APW – Prof. A Pathmeswaran – pathmes@kln.ac.lk
(University of Kelaniya, Sri Lanka)
ORCID ID 0000-0003-4065-2639

8) NML – Prof. Namal PM Liyanage - namal.malimbadaliyanage@osumc.edu
(Department of Microbial Infection and Immunity, College of Medicine, The Ohio State University, Ohio, United States)
ORCID ID: 0000-0001-7362-3282

9) ADW – Dr Ananda Wijewickrama - anandawijewickrama012@gmail.com
(Department of Medicine and Mental Health, Faculty of Medicine, University of Moratuwa, Sri Lanka)
ORCID ID: 0000-0003-2723-1813

10) ABM – Prof. U. Arjuna Bandara Medagama - arjuna.medagama@gmail.com
(Department of Medicine, Faculty of Medicine, University of Peradeniya, Sri Lanka)
ORCID ID: 0000-0002-8425-079X

Click here to access/download
Supporting Information
2 S1 PRISMA-P checklist Dengue SysR 28 May 2026.docx

Click here to access/download
Supporting Information
3. S1 Appendix Search Strategy Dengue SysR 28 May 2026.docx
