# Supplementary material for: Post-acute and long-term cardiovascular effects following dengue infection: A systematic review protocol
Source: PLoS One. 2026 Aug 21;21(8):e0356838. doi: 10.1371/journal.pone.0356838 (PMC13496405; doi:10.1371/journal.pone.0356838)
Supplement: Supplementary file 3 [file pone.0356838.s002.DOC]

**S1 Appendix: Search Strategy**

**1. PubMed/MEDLINE database search strategy**

The following draft PubMed/MEDLINE search strategy will be used and adapted for other databases, including Scopus, Cochrane Library, CINAHL, ScienceDirect, and ProQuest Dissertations and Theses, according to database-specific syntax.

(("Dengue"[MeSH] OR “Severe Dengue"[MeSH] OR “dengue” [Title/Abstract] OR "dengue hemorrhagic fever"[Title/Abstract] OR “Dengue Hemorrhagic Fever” [Title/Abstract] OR “DHF”

[Title/Abstract] OR "dengue infection"[Title/Abstract] OR "dengue virus"[MeSH])

AND

("Cardiovascular	Diseases"[MeSH]	OR	cardiovascular	[Title/Abstract]	OR	cardiac

[Title/Abstract] OR heart [Title/Abstract]

OR "heart failure"[Title/Abstract] OR "cardiac failure"[Title/Abstract]

OR "cardiac complication"[Title/Abstract] OR "cardiovascular complication"[Title/Abstract]

OR "cardiovascular sequela"[Title/Abstract] OR “Increased Carotid intima-Media Thickness”

[Title/Abstract] OR myocarditis [MeSH] OR myocarditis [Title/Abstract]

OR pericarditis [MeSH] OR pericarditis [Title/Abstract] OR myopericarditis [Title/Abstract]

OR "myocardial infarction"[MeSH] OR "acute coronary syndrome"[Title/Abstract]

OR arrhythmia [Title/Abstract] OR "cardiac arrhythmia"[Title/Abstract]

OR "atrial fibrillation"[MeSH] OR "atrial fibrillation"[Title/Abstract]

OR bradycardia [Title/Abstract] OR "sinus bradycardia"[Title/Abstract]

OR "heart block"[Title/Abstract] OR "atrioventricular block"[MeSH] OR "AV block"[Title/Abstract]

OR "conduction disorder"[Title/Abstract] OR "conduction block"[Title/Abstract]

OR stroke [MeSH] OR stroke [Title/Abstract] OR ischemic [Title/Abstract] OR ischaemic [Title/Abstract]

OR hemorrhagic [Title/Abstract] OR haemorrhagic [Title/Abstract]

OR TIA[Title/Abstract] OR "transient ischemic attack"[Title/Abstract]

OR “thromboembolism” [MeSH] OR “thromboembolism” [Title/Abstract] OR “thrombotic”

[Title/Abstract]

OR MACE [Title/Abstract]

OR “echocardiography” [MeSH] OR “echocardiography” [Title/Abstract])

AND

(“long-term"[Title/Abstract] OR long-term [Title/Abstract] OR chronic [Title/Abstract]

OR "post-acute"[Title/Abstract] OR follow-up [Title/Abstract] OR "follow up"[Title/Abstract] OR “History” [Title/Abstract] OR “longitudinal” [Title/Abstract] OR “cohort” [Title/Abstract] OR “survivors” [Title/Abstract]))

Limits/filters: Humans; publication period January 1985–March 2026. No language restriction will be applied at the search stage where feasible. Full-text eligibility assessment will be limited to articles published in English, Spanish, or Portuguese. Preprints will be excluded during screening where identifiable.

**2. Google Scholar Search Strategy**

Google Scholar will be searched as a supplementary grey-literature source rather than as a primary bibliographic database. The search will be conducted using predefined search phrases based on the three main concepts of the review: dengue infection, cardiovascular or cerebrovascular outcomes, and post-acute or long-term follow-up.

The following predefined Google Scholar search phrases will be used:

1. "dengue" "cardiovascular" "long-term"

2. "dengue" "cardiac" "follow-up"

3. "dengue" "cardiovascular sequelae"

4. "dengue" "post-acute" "cardiovascular"

5. "dengue" "heart failure" "cohort"

6. "dengue" "arrhythmia" "follow-up"

7. "dengue" "stroke" "long-term"

8. "dengue" "myocarditis" "follow-up"

9. "dengue" "endothelial dysfunction" "convalescent"

10. "dengue" "vascular" "biomarkers" "follow-up"

For each search phrase, up to the first 200 results sorted by relevance will be screened by title and snippet, unless relevance is exhausted earlier. Relevance will be considered exhausted if two consecutive Google Scholar results pages contain no potentially relevant records based on title and snippet screening. Records judged potentially eligible or unclear will be exported manually or recorded for further screening in Rayyan.

Google Scholar searches will be performed using a consistent browser/account approach where feasible. The search date, browser/account status, exact search phrase, number of results screened, reason for stopping if fewer than 200 results are screened, and potentially eligible records identified will be documented in a search log.

The eligibility criteria applied to Google Scholar records will be consistent with the main review protocol. Eligible records will be limited to human studies published from January 1985 to March 2026. Full-text eligibility assessment will be limited to articles published in English, Spanish, or Portuguese. Potentially relevant records in other languages will be documented separately but not fully assessed.
