# Supplementary material for: Post-acute and long-term cardiovascular effects following dengue infection: A systematic review protocol
Source: PLoS One. 2026 Aug 21;21(8):e0356838. doi: 10.1371/journal.pone.0356838 (PMC13496405; doi:10.1371/journal.pone.0356838)
Supplement: Supplementary file 4 [file pone.0356838.s005.DOC]

**Response to Reviewers: Post-acute and long-term cardiovascular effects following dengue infection: a systematic review protocol**

Dear Dr Liu,

We thank you and the reviewers for the careful evaluation of our manuscript, “Post-acute and long-term cardiovascular effects following dengue infection: a systematic review protocol” (PONE-D-26-25529). We have revised the manuscript in response to all journal requirements, reviewer comments, and Academic Editor comments. A point-by-point response is provided below. For this revised submission, we have uploaded the following files:

1. Response_to_reviewers_PONE-D-26-25529 – this file
2. Manuscript_PONE-D-26-25529 – clean revised manuscript
3. Revised_mansucript_with_track_changes_PONE-D-26-25529 – marked-up revised manuscript
4. S1_PRISMA-P_checklist_PONE-D-26-25529 – clean final version
5. S1_Appendix_Search_Strategy_PONE-D-26-25529 – clean final version

### Journal requirement (JR)

### Comment JR1. Please ensure that the manuscript meets PLOS ONE style requirements, including file naming.

**Response:** Thank you. We have checked the revised manuscript against the PLOS ONE formatting and file-naming requirements and have uploaded the required files as: “Response to Reviewers,” “Revised Manuscript with Track Changes,” and “Manuscript.”

### Comment JR2. Data Availability statement/contact point should not identify a manuscript author; revise the data statement and/or provide a non-author institutional contact.

**Response:** Thank you for identifying this inconsistency. This submission is a systematic review protocol; therefore, no datasets have yet been generated or analysed. We have revised the Data Availability statement to clarify that no data were generated or analysed for the current protocol and that, upon completion of the review, all data underlying the review findings will be made available without restriction as Supporting Information and/or through a public repository. We have removed the wording indicating availability from the corresponding author upon reasonable request and have corrected the online submission form accordingly. If an institutional contact is still required by the editorial office, we will provide a non-author institutional data access/ethics contact.

### Comment JR3. Reviewer-recommended citations should be evaluated for relevance; there is no requirement to cite them unless relevant.

**Response:** Thank you. No reviewer requested citation of a specific publication.

**Reviewer #1**

### Comment R1.1. Comparator group definition: provide a more precise definition of individuals without documented dengue infection and discuss how absence of dengue infection will be established, especially in endemic settings where asymptomatic infection is common.

**Response:** Thank you for this important comment. We agree that the definition of the comparator is critical, particularly in dengue-endemic settings where prior asymptomatic dengue infection may be common and not fully ascertainable.

We have revised the Comparator section to define comparator groups more precisely. Eligible comparators will include individuals without documented recent or index dengue infection, including laboratory-confirmed dengue-negative controls, non-dengue febrile illness controls, matched hospital or community controls, population controls, and other clearly defined comparison groups, as defined by the original studies.

We will extract how the absence of dengue infection was established in comparator participants, including laboratory testing, serology, clinical assessment, diagnostic coding, record review, or absence of documented dengue history. We will not require negative dengue serology for all comparator participants because many epidemiological studies in endemic settings may not have tested for previous dengue exposure. However, comparator ascertainment will be recorded and considered during risk-of-bias assessment and interpretation.

Where sufficient data are available, we will categorise studies according to comparator ascertainment: laboratory-confirmed dengue-negative controls; controls with no recorded or reported dengue history but without laboratory confirmation of dengue-negative status; and controls with unspecified or unclear dengue status. Where feasible, subgroup or sensitivity analyses will be conducted according to these categories. We have also added text discussing the potential for comparator exposure misclassification and its likely effect on the interpretation of comparative estimates.

### Comment R1.2. Coinfections and other viral illnesses: clarify whether studies involving chikungunya, Zika, influenza, SARS-CoV-2, or other arboviruses/viral coinfections will be excluded, analysed separately, or accounted for during extraction and synthesis.

**Response:** We agree that viral coinfections may independently contribute to cardiovascular manifestations and confound dengue-cardiovascular associations. We have revised the eligibility and data extraction sections to state that studies reporting dengue with confirmed or suspected coinfections will be included only if dengue-specific results are separable or if the coinfection status can be extracted and considered analytically. Studies in which cardiovascular outcomes cannot be attributed or separated for dengue-exposed participants will not contribute to primary comparative effect estimates. Coinfections will be extracted and considered through subgroup or sensitivity analyses where data permit; otherwise, they will be handled narratively as a limitation.

## Reviewer #2

### Comment R2.1. Exposure definition: dengue diagnosis needs standardisation. Restrict primary analyses to laboratory-confirmed dengue cases; include clinically diagnosed cases only in sensitivity or subgroup analyses; explicitly state how diagnostic heterogeneity will be handled.

**Response:** We thank the reviewer for highlighting this major source of potential bias. We have revised the Exposure section to define laboratory-confirmed dengue and to prioritise laboratory-confirmed dengue in primary comparative analyses. Studies based only on clearly stated clinical diagnostic criteria will remain eligible because this review aims to map the available evidence, but they will not be pooled with laboratory-confirmed studies in primary analyses unless diagnostic comparability is adequate. We will extract dengue confirmation methods in detail and consider exposure classification in ROBINS-E and in subgroup/sensitivity analyses.

### Comment R2.2. Comparator definition and misclassification bias: predefine subgroup analyses by laboratory-confirmed dengue-negative controls and population vs hospital controls; include sensitivity analyses excluding studies without confirmed dengue-negative comparators; discuss implications explicitly.

**Response:** This comment overlaps with Reviewer 1, Comment 1.1 regarding comparator definition and potential misclassification. In response, we revised the Comparator section to define eligible comparator groups more precisely and added details on how comparator dengue status will be extracted. We also added a planned analytical approach: where data permit, subgroup analyses will compare studies with laboratory-confirmed dengue-negative comparators against studies using population, hospital, community, or clinically defined comparator groups. We will also conduct sensitivity analyses excluding studies without confirmed dengue-negative comparator status, where feasible. The implications of possible undetected previous dengue infection among comparators, particularly in endemic settings, have been added to the risk-of-bias assessment and interpretation sections.

### Comment R2.3. Outcome heterogeneity and lack of standard definitions: predefine primary, secondary, and exploratory outcome categories; provide operational definitions where possible; clarify which outcomes will be eligible for meta-analysis.

**Response:** We thank the reviewer for this helpful suggestion. We have revised the Outcomes section to operationalise three tiers of outcomes: primary major clinical cardiovascular events, including cerebrovascular events such as stroke or transient ischaemic attack; secondary subclinical cardiac or vascular abnormalities; and exploratory cardiovascular-related biomarkers. We have also clarified that meta-analysis will be attempted only when outcome definitions, measurement methods, follow-up windows, comparator groups, and effect measures are sufficiently comparable; otherwise, findings will be synthesised narratively.

### Comment R2.4/R2.5. Definition of post-acute and long-term timeframes: justify the thresholds of 4 weeks to <3 months and ≥3 months.

**Response:** We agree that the timing thresholds should be explicitly justified. We have revised the Review Question and Scope section to clarify that the 4-week and 3-month thresholds are pragmatic review categories rather than strict biological boundaries. The 4-week threshold was selected to separate post-acute outcomes from acute-phase dengue manifestations and early convalescence, while the 3-month threshold was selected to distinguish longer-term follow-up from early post-acute findings. We have also clarified that original study timing definitions will be retained and that timing heterogeneity will be considered during synthesis and interpretation.

## Reviewer #3

### Comment R3.1. Statistical analysis plan: specify pooling strategies for different effect measures, random-effects estimators, and management of heterogeneous outcome definitions.

**Response:** We thank the reviewer for this helpful methodological comment. We have revised the Data Synthesis and Analysis section to provide a more explicit effect-pooling hierarchy and to clarify how heterogeneous outcome definitions and effect measures will be handled. We will preferentially extract adjusted effect estimates where available. Time-to-event estimates, risk ratios, odds ratios, and continuous outcome measures will not be pooled together unless conversion is methodologically appropriate and clinically meaningful. For continuous outcomes, mean differences will be used when measurement scales are comparable, and standardised mean differences will be used when scales differ. We have also specified that random-effects meta-analysis will use restricted maximum likelihood estimation for between-study variance, with appropriate caution when the number of studies is small. Outcomes will be pooled only when definitions, measurement methods, follow-up windows, comparator groups, and effect measures are sufficiently comparable; otherwise, findings will be synthesised narratively.

### Comment R3.2. Justify the rationale for language restrictions and temporal eligibility criteria.

**Response:** We thank the reviewer for this helpful suggestion. We have revised the Eligibility Criteria and Search Strategy sections to justify both the temporal and language eligibility criteria. The temporal criterion was selected to capture studies from the period of modern dengue epidemiology, diagnostic methods, and cardiovascular outcome reporting, while limiting older reports that may have limited applicability to current diagnostic and follow-up practice. The end date reflects the planned search period for the protocol.

We have also expanded language eligibility. Full-text articles published in English, Spanish, or Portuguese will be assessed for inclusion, as these languages are feasible for the review team and are relevant to dengue-endemic regions. Potentially relevant reports in other languages will be documented separately, but not fully assessed, and this will be acknowledged as a limitation of the review.

### Comment R3.3. Add detail regarding duplicate cohort management.

**Response:** We thank the reviewer for this important methodological suggestion. We have revised the Data Extraction section to clarify how multiple reports from the same or overlapping cohort will be handled. Reports arising from the same cohort, registry, database, or overlapping study population will be linked and treated as a single study for each outcome and follow-up time point to avoid double counting. The most comprehensive or methodologically appropriate report will be used for primary extraction, while additional reports will be used only for supplementary non-overlapping information, such as additional outcomes, longer follow-up, or subgroup data.

### Comment R3.4. Add reviewer calibration.

**Response:** We thank the reviewer for this useful suggestion. We have revised the Study Selection and Data Extraction sections to include a reviewer calibration procedure. Before formal screening, reviewers will pilot the eligibility criteria on a sample of retrieved records and discuss discrepancies to ensure consistent interpretation. Similarly, before full data extraction, reviewers will pilot the standardised extraction form on a small sample of eligible studies, resolve disagreements, and refine reviewer guidance before proceeding with independent extraction.

### Comment R3.5. Add detail regarding missing data handling.

**Response:** We have revised the protocol to specify how missing, incomplete, or unclear study-level data will be handled. Where key information required for eligibility assessment, risk-of-bias assessment, or synthesis is missing or unclear, we will attempt to contact study authors where feasible. If the information remains unavailable, it will be recorded as not reported or unclear. We will not impute missing outcome data. Summary statistics will be converted only when methodologically appropriate and when the required assumptions are sufficiently clear. The potential effect of missing data will be considered during risk-of-bias assessment and, where feasible, in sensitivity analyses.

### Comment R3.6. Improve reproducibility of Google Scholar searches.

**Response:** We thank the reviewer for this useful suggestion. We have revised the Search Strategy section to describe a predefined and reproducible approach for Google Scholar searching. Google Scholar will be used as a supplementary grey-literature source rather than as a primary bibliographic database. We have added details on predefined search phrases, search date, screening depth, and documentation in a search log.

### Comment R3.7. Acknowledge inherent limitations of observational evidence and potential residual confounding.

**Response:** We thank the reviewer for this important comment. We have revised the Data Synthesis and Analysis section to state that causal interpretation will be cautious because the expected evidence base is likely to be predominantly observational. We have added that residual confounding, exposure and comparator misclassification, surveillance bias, differential healthcare contact, and heterogeneous outcome definitions will be considered during risk-of-bias assessment, synthesis, GRADE certainty assessment, and interpretation.

## Academic Editor’s comments (marked in the manuscript):

### Comment AE1. Clarify the exposure definition.

**Response:** Addressed as described in response to Reviewer #2, Comment R2.1. We revised the Exposure section to prioritise laboratory-confirmed dengue in primary comparative synthesis and to handle clinically diagnosed dengue through descriptive, subgroup, or sensitivity analyses where appropriate.

### Comment AE2. Cite fragmentation; improve eligibility wording.

**Response:** Thank you. We have revised the manuscript to address both points. First, we added citations in the Introduction to support the statement that the existing evidence is fragmented because of differences in study design, population characteristics, dengue diagnostic criteria, comparator definitions, outcome definitions, and follow-up duration. Second, we revised the eligibility wording in the Abstract and Study Designs section for clarity, replacing “Eligible designs include…” with wording that makes it clear that studies using cohort, case-control, cross-sectional, or case-series designs will be eligible if they report relevant post-acute or long-term cardiovascular outcomes following dengue infection.

### Comment AE3. Avoid overstatement in objectives.

**Response:** Thank you. We have revised the Objectives section to avoid implying that the review can definitively determine causality or establish determinants of post-dengue cardiovascular risk. In the Primary Objective, we replaced the previous wording “determine the magnitude and determinants of post-dengue cardiovascular risk” with the more cautious phrase “estimate comparative cardiovascular risk where suitable data are available.” We also revised the comparative objective to use “estimate” rather than “compare,” and clarified that this will be done only where comparative data are available. In addition, we added “cerebrovascular events” to maintain consistency with the revised outcome framework. These revisions make the objectives more cautious and better aligned with the expected observational evidence base.

### Comment AE4. Justify post-acute cutoffs.

Response: Addressed as described in response to Reviewer #2, Comment R2.4/R2.5. We clarified that the 4-week and 3-month thresholds are pragmatic review categories rather than strict biological cut-offs.

### Comment AE5. Clarify comparator misclassification.

**Response:** Addressed as described in responses to Reviewer #1, Comment R1.1, and Reviewer #2, Comment R2.2. We expanded comparator definitions and added subgroup/sensitivity analysis plans for comparator dengue-negative confirmation and comparator source.

### Comment AE6. Justify language restrictions.

**Response:** Addressed together with Reviewer #3, Comment R3.2. We have revised the Eligibility Criteria and Search Strategy sections to justify the language eligibility criteria. In the revised protocol, full-text articles published in English, Spanish, or Portuguese will be assessed for inclusion, as these languages are feasible for the review team and are relevant to dengue-endemic regions, including Latin America and Brazil. Potentially relevant reports in other languages will be documented separately but not fully assessed, and the possibility of language-restriction bias will be acknowledged as a limitation of the review.

### Comment AE7. Define Google Scholar methodology.

**Response:** Addressed together with Reviewer #3, Comment R3.6. We have revised the Search Strategy section to classify Google Scholar as a supplementary grey literature source rather than a primary bibliographic database. We added details on predefined search phrases, screening depth, search date documentation, stopping rule, browser/account consistency where feasible, and search-log documentation. The predefined Google Scholar search phrases will also be reported in the search appendix.

### Comment AE8. Specify effect pooling hierarchy.

**Response:** Addressed as described in response to Reviewer #3, Comment R3.1. We added a hierarchy for adjusted estimates, effect measures, and handling of different designs and outcome types.

### Comment AE9. Add reviewer calibration.

**Response:** Addressed as described in response to Reviewer #3, Comment R3.4. We added calibration before formal screening and data extraction.

### Comment AE10. Specify random-effects estimator and GRADE details.

**Response:** Thank you. We have revised the Data Synthesis and Analysis section to specify the planned random-effects estimator. Between-study variance will be estimated using the restricted maximum likelihood (REML) estimator. We have also expanded the GRADE description to clarify that certainty will be assessed for main comparative outcomes where appropriate, considering risk of bias, inconsistency, indirectness, imprecision, and publication bias. We have added that upgrading will be considered only where justified, and that summary-of-findings tables will be prepared for main clinical outcomes where a coherent body of comparative evidence is available. Descriptive case-series evidence will generally not be subjected to formal GRADE assessment unless it contributes to a clearly defined body of evidence for a main outcome.

Sincerely,

Niroshan Chandrajith Lokunarangoda

Corresponding author, on behalf of all authors
[niroshanl@uom.lk](mailto:niroshanl@uom.lk)
