# Supplementary material for: Post-acute and long-term cardiovascular effects following dengue infection: A systematic review protocol
Source: PLoS One. 2026 Aug 21;21(8):e0356838. doi: 10.1371/journal.pone.0356838 (PMC13496405; doi:10.1371/journal.pone.0356838)
Supplement: Peer review history

# Peer review: Decision Letter 0

**DOI:** 10.1371/journal.pone.0356838.r001

**Authors:** Benjamin Liu

22 Jun 2026

Dear Dr. Lokunarangoda,

Thank you for submitting your manuscript to PLOS ONE. After careful consideration, we feel that it has merit but does not fully meet PLOS ONE’s publication criteria as it currently stands. Therefore, we invite you to submit a revised version of the manuscript that addresses the points raised during the review process.

Please submit your revised manuscript by Jul 22 2026 11:59PM. If you will need more time than this to complete your revisions, please reply to this message or contact the journal office at plosone@plos.org. When you're ready to submit your revision, log on to https://www.editorialmanager.com/pone/ and select the 'Submissions Needing Revision' folder to locate your manuscript file.

- A letter that responds to each point raised by the academic editor and reviewer(s). You should upload this letter as a separate file labeled 'Response to Reviewers'.
- A marked-up copy of your manuscript that highlights changes made to the original version. You should upload this as a separate file labeled 'Revised Manuscript with Track Changes'.
- An unmarked version of your revised paper without tracked changes. You should upload this as a separate file labeled 'Manuscript'.

If you would like to make changes to your financial disclosure, please include your updated statement in your cover letter. Guidelines for resubmitting your figure files are available below the reviewer comments at the end of this letter.

If applicable, we recommend that you deposit your laboratory protocols in protocols.io to enhance the reproducibility of your results. Protocols.io assigns your protocol its own identifier (DOI) so that it can be cited independently in the future. For instructions see: https://journals.plos.org/plosone/s/submission-guidelines#loc-laboratory-protocols. Additionally, PLOS ONE offers an option for publishing peer-reviewed Lab Protocol articles, which describe protocols hosted on protocols.io. Read more information on sharing protocols at https://plos.org/protocols?utm_medium=editorial-email&utm_source=authorletters&utm_campaign=protocols.

As the corresponding author, your ORCID iD is verified in the submission system and will appear in the published article. PLOS supports the use of ORCID, and we encourage all coauthors to register for an ORCID iD and use it as well. Please encourage your coauthors to verify their ORCID iD within the submission system before final acceptance, as unverified ORCID iDs will not appear in the published article. *Only*  the individual author can complete the verification step; PLOS staff *cannot*  verify ORCID iDs on behalf of authors.

We look forward to receiving your revised manuscript.

Kind regards,

Benjamin M. Liu, PhD, D(ABMM), MB(ASCP)

Academic Editor

PLOS One

**Journal Requirements:**

When submitting your revision, we need you to address these additional requirements.

1. Please ensure that your manuscript meets PLOS ONE's style requirements, including those for file naming. The PLOS ONE style templates can be found at

https://journals.plos.org/plosone/s/file?id=wjVg/PLOSOne_formatting_sample_main_body.pdf and

https://journals.plos.org/plosone/s/file?id=ba62/PLOSOne_formatting_sample_title_authors_affiliations.pdf

2. In the online submission form you indicate that your data is not available for proprietary reasons and have provided a contact point for accessing this data. Please note that your current contact point is a co-author on this manuscript. According to our Data Policy, the contact point must not be an author on the manuscript and must be an institutional contact, ideally not an individual. Please revise your data statement to a non-author institutional point of contact, such as a data access or ethics committee, and send this to us via return email. Please also include contact information for the third party organization, and please include the full citation of where the data can be found.

3. If the reviewer comments include a recommendation to cite specific previously published works, please review and evaluate these publications to determine whether they are relevant and should be cited. There is no requirement to cite these works unless the editor has indicated otherwise.

[Note: HTML markup is below. Please do not edit.]

Reviewers' comments:

Reviewer's Responses to Questions

**Comments to the Author**

1. Does the manuscript provide a valid rationale for the proposed study, with clearly identified and justified research questions?

Reviewer #1: Yes

Reviewer #2: Yes

Reviewer #3: Yes

**********

2. Is the protocol technically sound and planned in a manner that will lead to a meaningful outcome and allow testing the stated hypotheses?

Reviewer #1: Yes

Reviewer #2: Yes

Reviewer #3: Yes

**********

3. Is the methodology feasible and described in sufficient detail to allow the work to be replicable?

Reviewer #1: Yes

Reviewer #2: No

Reviewer #3: Yes

**********

4. Have the authors described where all data underlying the findings will be made available when the study is complete?

The PLOS Data policy

Reviewer #1: Yes

Reviewer #2: Yes

Reviewer #3: Yes

**********

5. Is the manuscript presented in an intelligible fashion and written in standard English?

Reviewer #1: Yes

Reviewer #2: Yes

Reviewer #3: Yes

**********

Please use the space provided to explain your answers to the questions above and, if applicable, provide comments about issues authors must address before this protocol can be accepted for publication. You may also include additional comments for the author, including concerns about research or publication ethics.

You may also provide optional suggestions and comments to authors that they might find helpful in planning their study.

Reviewer #1: Thank you for the opportunity to review this protocol. I have the following comments for the authors' consideration:

Definition of the Comparator Group

The comparator group is described as individuals without documented dengue infection. Please provide a more precise definition of this group. Specifically, how will the absence of dengue infection be established in the included studies? Will comparator participants be required to have negative dengue serology or other laboratory evidence excluding prior dengue infection, particularly in endemic settings where asymptomatic infections are common? The potential for misclassification should be discussed.

Coinfections and Other Viral Illnesses

Viral coinfections may independently contribute to cardiovascular manifestations and could confound observed associations. Please clarify whether studies involving coinfection with other viruses (e.g., chikungunya, Zika, influenza, SARS-CoV-2, or other arboviruses) will be excluded, analyzed separately, or accounted for during data extraction and synthesis.

Addressing these points would improve the methodological clarity and validity of the planned review.

Reviewer #2: This protocol addresses an important and timely public health question on the long-term cardiovascular sequelae of dengue infection. The manuscript is generally well-organized, adheres to PRISMA-P guidelines, and demonstrates thoughtful planning (e.g., PROSPERO registration, use of ROBINS-E, GRADE framework).

However, several critical methodological issues require clarification and strengthening to ensure rigor, reproducibility, and validity of the eventual review. These relate primarily to exposure definition, comparator misclassification, heterogeneity management, and analytical strategy.

I recommend major revision prior to acceptance.

Major Comments

1. Exposure Definition (Dengue Diagnosis Needs Standardization)

The protocol allows inclusion of studies with dengue diagnosis based on:

“laboratory, serological, molecular, or clearly stated clinical diagnostic criteria”

Concern:

Clinical diagnosis of dengue varies widely across settings and may introduce substantial exposure misclassification bias, particularly in retrospective or resource-limited settings.

Recommendation:

• Restrict primary analyses to laboratory-confirmed dengue cases (PCR, NS1 antigen, IgM serology).

• Include clinically diagnosed cases only in:

o Sensitivity analyses

o Or clearly defined subgroup analyses

• Explicitly state how diagnostic heterogeneity will be handled during synthesis.

2. Comparator Definition and Misclassification Bias

The authors acknowledge that:

“absence of any previous dengue infection may not be fully ascertainable”

Concern (Critical):

In endemic regions, comparator groups may include individuals with prior undetected dengue infection, leading to non-differential misclassification and bias toward the null.

Missing:

No analytical strategy to mitigate this issue is described.

Recommendation:

• Predefine subgroup analyses based on:

o Laboratory-confirmed dengue-negative controls

o Population vs hospital controls

• Include sensitivity analyses excluding studies without confirmed dengue-negative comparators

• Discuss implications explicitly in protocol methods.

3. Outcome Heterogeneity and Lack of Standard Definitions

The protocol includes a broad range of outcomes:

• Clinical events, imaging abnormalities, and biomarkers

Concern:

This introduces substantial clinical heterogeneity, and:

• No standardized definitions are provided

• No strict prioritization scheme is operationalized

Recommendation:

• Predefine outcome categories:

o Primary outcomes: Major cardiovascular events (e.g., MI, stroke, HF)

o Secondary outcomes: Subclinical/imaging findings

o Exploratory outcomes: Biomarkers

• Provide operational definitions where possible

• Clarify which outcomes will be eligible for meta-analysis

5. Definition of Post-acute and Long-term Timeframes

The protocol defines:

• Post-acute: 4 weeks to <3 months

• Long-term: ≥3 months

Concern:

These cut-offs appear arbitrary and not biologically justified, and studies may use heterogeneous follow-up intervals.

Recommendation:

• Provide justification for chosen thresholds

Reviewer #3: This manuscript presents a protocol for a systematic review investigating post-acute and long-term cardiovascular sequelae following dengue infection, an increasingly recognized area of clinical importance. The topic is timely, highly relevant to global health, and has substantial implications for cardiovascular surveillance and long-term management in dengue-endemic regions. The protocol is generally well organized and demonstrates careful planning through adherence to PRISMA-P reporting standards, prospective PROSPERO registration, predefined eligibility criteria, structured data extraction procedures, and appropriate use of ROBINS-E, JBI critical appraisal tools, and GRADE methodology. These features indicate a strong methodological foundation.

The principal strengths include the comprehensive scope of cardiovascular outcomes, inclusion of both clinical and subclinical manifestations, and planned synthesis of biomarker evidence alongside major cardiovascular events. The multidisciplinary review team and planned dual independent screening and extraction procedures further strengthen methodological quality.

Several areas, however, require clarification before publication. The statistical analysis plan should specify pooling strategies for different effect measures, random-effects estimators, and management of heterogeneous outcome definitions. The rationale for language restrictions and temporal eligibility criteria should be justified explicitly. Additional detail regarding duplicate cohort management, reviewer calibration, missing data handling, and reproducibility of Google Scholar searches would improve transparency. Finally, the protocol should acknowledge the inherent limitations of observational evidence and potential residual confounding when interpreting associations.

Overall, this is a high-quality systematic review protocol addressing an important clinical question.

**********

what does this mean?). If published, this will include your full peer review and any attached files.

If you choose “no”, your identity will remain anonymous but your review may still be made public.

**Do you want your identity to be public for this peer review?**  For information about this choice, including consent withdrawal, please see our Privacy Policy

Reviewer #1: **Yes:** Vithiya Ganesan

Reviewer #2: **Yes:** Kshitiz Gupta

Reviewer #3: No

**********

[NOTE: If reviewer comments were submitted as an attachment file, they will be attached to this email and accessible via the submission site. Please log into your account, locate the manuscript record, and check for the action link "View Attachments". If this link does not appear, there are no attachment files.]

To ensure your figures meet our technical requirements, please review our figure guidelines: https://journals.plos.org/plosone/s/figures

You may also use PLOS’s free figure tool, NAAS, to help you prepare publication quality figures: https://journals.plos.org/plosone/s/figures#loc-tools-for-figure-preparation.

NAAS will assess whether your figures meet our technical requirements by comparing each figure against our figure specifications.

Attachment Submitted filename: Reviewed_Protocol_with_Comments.pdf

# Author comment: Author response to Decision Letter 1

**DOI:** 10.1371/journal.pone.0356838.r002

16 Jul 2026

Journal requirement (JR)

Comment JR1. Please ensure that the manuscript meets PLOS ONE style requirements, including file naming.

Response: Thank you. We have checked the revised manuscript against the PLOS ONE formatting and file-naming requirements and have uploaded the required files as: “Response to Reviewers,” “Revised Manuscript with Track Changes,” and “Manuscript.”

Comment JR2. Data Availability statement/contact point should not identify a manuscript author; revise the data statement and/or provide a non-author institutional contact.

Response: Thank you for identifying this inconsistency. This submission is a systematic review protocol; therefore, no datasets have yet been generated or analysed. We have revised the Data Availability statement to clarify that no data were generated or analysed for the current protocol and that, upon completion of the review, all data underlying the review findings will be made available without restriction as Supporting Information and/or through a public repository. We have removed the wording indicating availability from the corresponding author upon reasonable request and have corrected the online submission form accordingly. If an institutional contact is still required by the editorial office, we will provide a non-author institutional data access/ethics contact.

Comment JR3. Reviewer-recommended citations should be evaluated for relevance; there is no requirement to cite them unless relevant.

Response: Thank you. No reviewer requested citation of a specific publication.

Reviewer #1

Comment R1.1. Comparator group definition: provide a more precise definition of individuals without documented dengue infection and discuss how absence of dengue infection will be established, especially in endemic settings where asymptomatic infection is common.

Response: Thank you for this important comment. We agree that the definition of the comparator is critical, particularly in dengue-endemic settings where prior asymptomatic dengue infection may be common and not fully ascertainable.

We have revised the Comparator section to define comparator groups more precisely. Eligible comparators will include individuals without documented recent or index dengue infection, including laboratory-confirmed dengue-negative controls, non-dengue febrile illness controls, matched hospital or community controls, population controls, and other clearly defined comparison groups, as defined by the original studies.

We will extract how the absence of dengue infection was established in comparator participants, including laboratory testing, serology, clinical assessment, diagnostic coding, record review, or absence of documented dengue history. We will not require negative dengue serology for all comparator participants because many epidemiological studies in endemic settings may not have tested for previous dengue exposure. However, comparator ascertainment will be recorded and considered during risk-of-bias assessment and interpretation.

Where sufficient data are available, we will categorise studies according to comparator ascertainment: laboratory-confirmed dengue-negative controls; controls with no recorded or reported dengue history but without laboratory confirmation of dengue-negative status; and controls with unspecified or unclear dengue status. Where feasible, subgroup or sensitivity analyses will be conducted according to these categories. We have also added text discussing the potential for comparator exposure misclassification and its likely effect on the interpretation of comparative estimates.

Comment R1.2. Coinfections and other viral illnesses: clarify whether studies involving chikungunya, Zika, influenza, SARS-CoV-2, or other arboviruses/viral coinfections will be excluded, analysed separately, or accounted for during extraction and synthesis.

Response: We agree that viral coinfections may independently contribute to cardiovascular manifestations and confound dengue-cardiovascular associations. We have revised the eligibility and data extraction sections to state that studies reporting dengue with confirmed or suspected coinfections will be included only if dengue-specific results are separable or if the coinfection status can be extracted and considered analytically. Studies in which cardiovascular outcomes cannot be attributed or separated for dengue-exposed participants will not contribute to primary comparative effect estimates. Coinfections will be extracted and considered through subgroup or sensitivity analyses where data permit; otherwise, they will be handled narratively as a limitation.

Reviewer #2

Comment R2.1. Exposure definition: dengue diagnosis needs standardisation. Restrict primary analyses to laboratory-confirmed dengue cases; include clinically diagnosed cases only in sensitivity or subgroup analyses; explicitly state how diagnostic heterogeneity will be handled.

Response: We thank the reviewer for highlighting this major source of potential bias. We have revised the Exposure section to define laboratory-confirmed dengue and to prioritise laboratory-confirmed dengue in primary comparative analyses. Studies based only on clearly stated clinical diagnostic criteria will remain eligible because this review aims to map the available evidence, but they will not be pooled with laboratory-confirmed studies in primary analyses unless diagnostic comparability is adequate. We will extract dengue confirmation methods in detail and consider exposure classification in ROBINS-E and in subgroup/sensitivity analyses.

Comment R2.2. Comparator definition and misclassification bias: predefine subgroup analyses by laboratory-confirmed dengue-negative controls and population vs hospital controls; include sensitivity analyses excluding studies without confirmed dengue-negative comparators; discuss implications explicitly.

Response: This comment overlaps with Reviewer 1, Comment 1.1 regarding comparator definition and potential misclassification. In response, we revised the Comparator section to define eligible comparator groups more precisely and added details on how comparator dengue status will be extracted. We also added a planned analytical approach: where data permit, subgroup analyses will compare studies with laboratory-confirmed dengue-negative comparators against studies using population, hospital, community, or clinically defined comparator groups. We will also conduct sensitivity analyses excluding studies without confirmed dengue-negative comparator status, where feasible. The implications of possible undetected previous dengue infection among comparators, particularly in endemic settings, have been added to the risk-of-bias assessment and interpretation sections.

Comment R2.3. Outcome heterogeneity and lack of standard definitions: predefine primary, secondary, and exploratory outcome categories; provide operational definitions where possible; clarify which outcomes will be eligible for meta-analysis.

Response: We thank the reviewer for this helpful suggestion. We have revised the Outcomes section to operationalise three tiers of outcomes: primary major clinical cardiovascular events, including cerebrovascular events such as stroke or transient ischaemic attack; secondary subclinical cardiac or vascular abnormalities; and exploratory cardiovascular-related biomarkers. We have also clarified that meta-analysis will be attempted only when outcome definitions, measurement methods, follow-up windows, comparator groups, and effect measures are sufficiently comparable; otherwise, findings will be synthesised narratively.

Comment R2.4/R2.5. Definition of post-acute and long-term timeframes: justify the thresholds of 4 weeks to <3 months and ≥3 months.

Response: We agree that the timing thresholds should be explicitly justified. We have revised the Review Question and Scope section to clarify that the 4-week and 3-month thresholds are pragmatic review categories rather than strict biological boundaries. The 4-week threshold was selected to separate post-acute outcomes from acute-phase dengue manifestations and early convalescence, while the 3-month threshold was selected to distinguish longer-term follow-up from early post-acute findings. We have also clarified that original study timing definitions will be retained and that timing heterogeneity will be considered during synthesis and interpretation.

Reviewer #3

Comment R3.1. Statistical analysis plan: specify pooling strategies for different effect measures, random-effects estimators, and management of heterogeneous outcome definitions.

Response: We thank the reviewer for this helpful methodological comment. We have revised the Data Synthesis and Analysis section to provide a more explicit effect-pooling hierarchy and to clarify how heterogeneous outcome definitions and effect measures will be handled. We will preferentially extract adjusted effect estimates where available. Time-to-event estimates, risk ratios, odds ratios, and continuous outcome measures will not be pooled together unless conversion is methodologically appropriate and clinically meaningful. For continuous outcomes, mean differences will be used when measurement scales are comparable, and standardised mean differences will be used when scales differ. We have also specified that random-effects meta-analysis will use restricted maximum likelihood estimation for between-study variance, with appropriate caution when the number of studies is small. Outcomes will be pooled only when definitions, measurement methods, follow-up windows, comparator groups, and effect measures are sufficiently comparable; otherwise, findings will be synthesised narratively.

Comment R3.2. Justify the rationale for language restrictions and temporal eligibility criteria.

Response: We thank the reviewer for this helpful suggestion. We have revised the Eligibility Criteria and Search Strategy sections to justify both the temporal and language eligibility criteria. The temporal criterion was selected to capture studies from the period of modern dengue epidemiology, diagnostic methods, and cardiovascular outcome reporting, while limiting older reports that may have limited applicability to current diagnostic and follow-up practice. The end date reflects the planned search period for the protocol.

We have also expanded language eligibility. Full-text articles published in English, Spanish, or Portuguese will be assessed for inclusion, as these languages are feasible for the review team and are relevant to dengue-endemic regions. Potentially relevant reports in other languages will be documented separately, but not fully assessed, and this will be acknowledged as a limitation of the review.

Comment R3.3. Add detail regarding duplicate cohort management.

Response: We thank the reviewer for this important methodological suggestion. We have revised the Data Extraction section to clarify how multiple reports from the same or overlapping cohort will be handled. Reports arising from the same cohort, registry, database, or overlapping study population will be linked and treated as a single study for each outcome and follow-up time point to avoid double counting. The most comprehensive or methodologically appropriate report will be used for primary extraction, while additional reports will be used only for supplementary non-overlapping information, such as additional outcomes, longer follow-up, or subgroup data.

Comment R3.4. Add reviewer calibration.

Response: We thank the reviewer for this useful suggestion. We have revised the Study Selection and Data Extraction sections to include a reviewer calibration procedure. Before formal screening, reviewers will pilot the eligibility criteria on a sample of retrieved records and discuss discrepancies to ensure consistent interpretation. Similarly, before full data extraction, reviewers will pilot the standardised extraction form on a small sample of eligible studies, resolve disagreements, and refine reviewer guidance before proceeding with independent extraction.

Comment R3.5. Add detail regarding missing data handling.

Response: We have revised the protocol to specify how missing, incomplete, or unclear study-level data will be handled. Where key information required for eligibility assessment, risk-of-bias assessment, or synthesis is missing or unclear, we will attempt to contact study authors where feasible. If the information remains unavailable, it will be recorded as not reported or unclear. We will not impute missing outcome data. Summary statistics will be converted only when methodologically appropriate and when the required assumptions are sufficiently clear. The potential effect of missing data will be considered during risk-of-bias assessment and, where feasible, in sensitivity analyses.

Comment R3.6. Improve reproducibility of Google Scholar searches.

Response: We thank the reviewer for this useful suggestion. We have revised the Search Strategy section to describe a predefined and reproducible approach for Google Scholar searching. Google Scholar will be used as a supplementary grey-literature source rather than as a primary bibliographic database. We have added details on predefined search phrases, search date, screening depth, and documentation in a search log.

Comment R3.7. Acknowledge inherent limitations of observational evidence and potential residual confounding.

Response: We thank the reviewer for this important comment. We have revised the Data Synthesis and Analysis section to state that causal interpretation will be cautious because the expected evidence base is likely to be predominantly observational. We have added that residual confounding, exposure and comparator misclassification, surveillance bias, differential healthcare contact, and heterogeneous outcome definitions will be considered during risk-of-bias assessment, synthesis, GRADE certainty assessment, and interpretation.

Academic Editor’s comments (marked in the manuscript):

Comment AE1. Clarify the exposure definition.

Response: Addressed as described in response to Reviewer #2, Comment R2.1. We revised the Exposure section to prioritise laboratory-confirmed dengue in primary comparative synthesis and to handle clinically diagnosed dengue through descriptive, subgroup, or sensitivity analyses where appropriate.

Comment AE2. Cite fragmentation; improve eligibility wording.

Response: Thank you. We have revised the manuscript to address both points. First, we added citations in the Introduction to support the statement that the existing evidence is fragmented because of differences in study design, population characteristics, dengue diagnostic criteria, comparator definitions, outcome definitions, and follow-up duration. Second, we revised the eligibility wording in the Abstract and Study Designs section for clarity, replacing “Eligible designs include…” with wording that makes it clear that studies using cohort, case-control, cross-sectional, or case-series designs will be eligible if they report relevant post-acute or long-term cardiovascular outcomes following dengue infection.

Comment AE3. Avoid overstatement in objectives.

Response: Thank you. We have revised the Objectives section to avoid implying that the review can definitively determine causality or establish determinants of post-dengue cardiovascular risk. In the Primary Objective, we replaced the previous wording “determine the magnitude and determinants of post-dengue cardiovascular risk” with the more cautious phrase “estimate comparative cardiovascular risk where suitable data are available.” We also revised the comparative objective to use “estimate” rather than “compare,” and clarified that this will be done only where comparative data are available. In addition, we added “cerebrovascular events” to maintain consistency with the revised outcome framework. These revisions make the objectives more cautious and better aligned with the expected observational evidence base.

Comment AE4. Justify post-acute cutoffs.

Response: Addressed as described in response to Reviewer #2, Comment R2.4/R2.5. We clarified that the 4-week and 3-month thresholds are pragmatic review categories rather than strict biological cut-offs.

Comment AE5. Clarify comparator misclas

Attachment Submitted filename: Response_to_reviewers_PONE-D-26-25529.DOC

# Peer review: Decision Letter 1

**DOI:** 10.1371/journal.pone.0356838.r003

**Authors:** Benjamin Liu

10 Aug 2026

Post-acute and long-term cardiovascular effects following dengue infection: a systematic review protocol

PONE-D-26-25529R1

Dear Dr. Lokunarangoda,

We’re pleased to inform you that your manuscript has been judged scientifically suitable for publication and will be formally accepted for publication once it meets all outstanding technical requirements.

Within one week, you’ll receive an e-mail detailing the required amendments. When these have been addressed, you’ll receive a formal acceptance letter and your manuscript will be scheduled for publication.

An invoice will be generated when your article is formally accepted. Please note, if your institution has a publishing partnership with PLOS and your article meets the relevant criteria, all or part of your publication costs will be covered. Please make sure your user information is up-to-date by logging into Editorial Manager at Editorial Manager® and clicking the ‘Update My Information' link at the top of the page. For questions related to billing, please contact billing support.

If your institution or institutions have a press office, please notify them about your upcoming paper to help maximize its impact. If they’ll be preparing press materials, please inform our press team as soon as possible -- no later than 48 hours after receiving the formal acceptance. Your manuscript will remain under strict press embargo until 2 pm Eastern Time on the date of publication. For more information, please contact onepress@plos.org.

Kind regards,

Benjamin M. Liu, PhD, D(ABMM), MB(ASCP)

Academic Editor

PLOS One

Additional Editor Comments (optional):

Reviewers' comments:

Reviewer's Responses to Questions

**Comments to the Author**

1. Does the manuscript provide a valid rationale for the proposed study, with clearly identified and justified research questions?

Reviewer #1: Yes

Reviewer #3: Yes

**********

2. Is the protocol technically sound and planned in a manner that will lead to a meaningful outcome and allow testing the stated hypotheses?

Reviewer #1: Yes

Reviewer #3: Yes

**********

3. Is the methodology feasible and described in sufficient detail to allow the work to be replicable?

Reviewer #1: Yes

Reviewer #3: Yes

**********

4. Have the authors described where all data underlying the findings will be made available when the study is complete?

The PLOS Data policy

Reviewer #1: Yes

Reviewer #3: Yes

**********

5. Is the manuscript presented in an intelligible fashion and written in standard English?

Reviewer #1: Yes

Reviewer #3: Yes

**********

Please use the space provided to explain your answers to the questions above and, if applicable, provide comments about issues authors must address before this protocol can be accepted for publication. You may also include additional comments for the author, including concerns about research or publication ethics.

You may also provide optional suggestions and comments to authors that they might find helpful in planning their study.

Reviewer #1: All queries have been addressed appropriately. The revised protocol is clear, scientifically sound, and ethically appropriate. The authors have satisfactorily responded to the review comments, and no major concerns remain. I have no further comments or revisions to suggest. The protocol is suitable for acceptance for publication in its current form.

Reviewer #3: This is a PRISMA-P–compliant protocol for a systematic review of cardiovascular and cerebrovascular outcomes occurring at least four weeks after acute dengue infection. The question is timely and clinically important: the two anchor cohort studies from Taiwan and Singapore, together with the echocardiographic literature, have generated a genuine signal that the field has not yet synthesised, and the global burden data cited by the authors make the case for doing so. The protocol is registered, the team is multidisciplinary and appropriately constituted, and the reporting structure is broadly sound.

The first-round revision is substantial and, in several respects, genuinely responsive. The exposure hierarchy (laboratory-confirmed versus clinically diagnosed dengue), the tiered outcome framework, the comparator ascertainment categories, reviewer calibration, duplicate-cohort linkage, and missing-data handling are all materially improved. My recommendation for major revision should not be read as a negative judgement of the underlying project.

It reflects, rather, three things. First, several of the previous round’s comments have been answered in the response letter more completely than in the manuscript itself. Second, the revision has introduced new problems that were not present in the original submission, including the loss of citation searching and a divergence between the clean and marked-up files. Third, and most importantly, a set of methodological issues that are decisive for this particular literature — time-zero definition, the eligibility of follow-up windows that straddle the four-week threshold, the temporality of cross-sectional serosurveys, and detection bias — are not addressed anywhere in the protocol. Because a protocol’s entire value lies in committing to these decisions before the data are seen, they cannot be deferred to the review itself.

**********

what does this mean?). If published, this will include your full peer review and any attached files.

If you choose “no”, your identity will remain anonymous but your review may still be made public.

**Do you want your identity to be public for this peer review?**  For information about this choice, including consent withdrawal, please see our Privacy Policy

Reviewer #1: **Yes:** VITHIYA GANESAN

Reviewer #3: No

**********

# Editor report: Acceptance letter

**DOI:** 10.1371/journal.pone.0356838.r004

**Authors:** Benjamin Liu

PONE-D-26-25529R1

PLOS One

Dear Dr. Lokunarangoda,

I'm pleased to inform you that your manuscript has been deemed suitable for publication in PLOS One. Congratulations! Your manuscript is now being handed over to our production team.

At this stage, our production department will prepare your paper for publication. This includes ensuring the following:

* All references, tables, and figures are properly cited

* All relevant supporting information is included in the manuscript submission,

* There are no issues that prevent the paper from being properly typeset

You will receive further instructions from the production team, including instructions on how to review your proof when it is ready. Please keep in mind that we are working through a large volume of accepted articles, so please give us a few days to review your paper and let you know the next and final steps.

Lastly, if your institution or institutions have a press office, please let them know about your upcoming paper now to help maximize its impact. If they'll be preparing press materials, please inform our press team within the next 48 hours. Your manuscript will remain under strict press embargo until 2 pm Eastern Time on the date of publication. For more information, please contact onepress@plos.org.

You will receive an invoice from PLOS for your publication fee after your manuscript has reached the completed accept phase. If you receive an email requesting payment before acceptance or for any other service, this may be a phishing scheme. Learn how to identify phishing emails and protect your accounts at https://explore.plos.org/phishing.

If we can help with anything else, please email us at customercare@plos.org.

Thank you for submitting your work to PLOS One and supporting open access.

Kind regards,

PLOS ONE Editorial Office Staff

on behalf of

Dr. Benjamin M. Liu

Academic Editor

PLOS One
